# Cisplatin Resistance in Testicular Germ Cell Tumors: Current Challenges from Various Perspectives

**DOI:** 10.3390/cancers12061601

**Published:** 2020-06-17

**Authors:** João Lobo, Carmen Jerónimo, Rui Henrique

**Affiliations:** 1 Cancer Biology and Epigenetics Group, IPO Porto Research Center (GEBC CI-IPOP), Portuguese Oncology Institute of Porto (IPO Porto) & Porto Comprehensive Cancer Center (P.CCC), R. Dr. António Bernardino de Almeida, 4200-072 Porto, Portugal; jpedro.lobo@ipoporto.min-saude.pt; 2 Department of Pathology, Portuguese Oncology Institute of Porto (IPOP), R. Dr. António Bernardino de Almeida, 4200-072 Porto, Portugal; 3 Department of Pathology and Molecular Immunology, Institute of Biomedical Sciences Abel Salazar, University of Porto (ICBAS-UP), Rua Jorge Viterbo Ferreira 228, 4050-513 Porto, Portugal

**Keywords:** testicular germ-cell tumors, cisplatin, resistance, targeted treatment

## Abstract

Testicular germ cell tumors share a marked sensitivity to cisplatin, contributing to their overall good prognosis. However, a subset of patients develop resistance to platinum-based treatments, by still-elusive mechanisms, experiencing poor quality of life due to multiple (often ineffective) interventions and, eventually, dying from disease. Currently, there is a lack of defined treatment opportunities for these patients that tackle the mechanism(s) underlying the emergence of resistance. Herein, we aim to provide a multifaceted overview of cisplatin resistance in testicular germ cell tumors, from the clinical perspective, to the pathobiology (including mechanisms contributing to induction of the resistant phenotype), to experimental models available for studying this occurrence. We provide a systematic summary of pre-target, on-target, post-target, and off-target mechanisms putatively involved in cisplatin resistance, providing data from preclinical studies and from those attempting validation in clinical samples, including those exploring specific alterations as therapeutic targets, some of them included in ongoing clinical trials. We briefly discuss the specificities of resistance related to teratoma (differentiated) phenotype, including the phenomena of growing teratoma syndrome and development of somatic-type malignancy. Cisplatin resistance is most likely multifactorial, and a combination of therapeutic strategies will most likely produce the best clinical benefit.

## 1. Introduction: A Brief Overview over Testicular Germ Cell Tumors

Testicular germ cell tumors (TGCTs) are the most frequent solid neoplasms arising in men aged until 34 years, and their incidence is rising worldwide [1]. This is due to environmental risk factors, together with a genetic contribution (the “genvironment” model) [2]. These tumors are part of the broader family of germ cell tumors (GCTs), remarkable for their link to developmental biology [2,3]. Among GCTs, which include also ovarian and extragonadal tumors of all age groups (pediatric and adult), type II TGCTs stand out as the most frequent and clinically challenging due to their malignant behavior. They have a unique pathobiology, deriving from primordial germ cells (PGCs)/gonocytes that are arrested in their maturation, and evolve towards a precursor neoplastic lesion, germ cell neoplasia in situ (GCNIS) [4]. This lesion is in the center of the 2016 World Health Organization classification for these tumors [5] and will almost invariably progress into invasive cancer, which histologically and clinically is divided into two distinct subgroups, with different prognosis and response to treatment: seminomas, the neoplastic resemblance of PGCs, and the nonseminomas. The latter, frequently more aggressive, are heterogeneous, including both embryonal carcinoma (the neoplastic resemblance of embryonic stem cells—ESCs), the extraembryonic derived choriocarcinoma and yolk sac tumor, and the most differentiated somatic-like teratoma [5].

TGCTs represent a unique model of curable cancer [6], which is mostly due to introduction of cisplatin, in the 1970s, that changed the paradigm of patient care [7]. Currently, survival rates frequently surpass 95%, and even patients relapsing or presenting disseminated disease are effectively treated with this agent, given in several successive treatment courses, if needed [8]. Since, at present, 70% of patients will be diagnosed with the localized disease only [9], efforts should be directed towards uncovering biomarkers (like vascular invasion [10]) that discriminate patients that will relapse and thus deserve adjuvant systemic treatment after surgery from those that are cured by orchiectomy alone and can avoid the long-term side effects of this compound and that are already reported to impact the quality of life of young-adult cancer survivors [11,12,13]. In other words, avoiding overtreatment should be a major aim of current clinical research; however, on the other side of the spectrum, there are TGCT that develop resistance to cisplatin. This is an uncommon event but long recognized and clinically meaningful, since there is a lack of alternative treatments for these patients, which are the ones experiencing important morbidity (and mortality) [14]. Study of this phenomenon has pointed towards several implicated mechanisms but, to date, a definitive culprit is missing, and biomarkers for predicting its emergence are still lacking, despite recent promising breakthroughs [15,16,17,18,19,20,21,22,23].

In this review, we sought to briefly present the clinical scenario of a cisplatin-resistant disease in TGCT, critically summarize the various mechanisms potentially involved in this event, indicate which tools are available for illuminating this event, and, finally, approach some promising therapeutic options for these patients.

## 2. The Clinical Impact of a Cisplatin-Resistant Disease

Even upon metastatic dissemination, TGCT cure rates may go up to 90% [18]. For those patients that relapse (around 30%, particularly common in those with poor prognostic features at diagnosis) [8], still 50% of these will achieve complete remission upon a salvage, multimodal chemotherapy, meaning that the proportion of GCT patients actually completely failing to respond to platinum-based treatment is only 3–5% [18,24]. This is, however, the subset of patients with cisplatin-resistant disease that end up dying of their disease within a few months. Clinically, a platinum-refractory disease has been defined (although with variations depending on the study) as a disease that continually progresses under chemotherapy, that relapses/progresses after second-line platinum-based chemotherapy, or that responds/remains stable during the initial cisplatin-based treatment but relapses within one month of completing it [8,18]. In practice, definitions of a response to chemotherapy can be envisioned from several perspectives, both clinical complete remission (with negative imaging scans and normalization of alpha-fetoprotein (AFP) and human chorionic gonadotropin (HCG) serum levels), surgical complete remission (implying the absence of residual macroscopic disease after the surgical procedure), or pathological complete remission (allowing the presence of necrosis/fibrosis or mature teratoma but no evidence of a nonteratoma viable disease), all of which bring distinct inputs to clinical decision-making [25]. 

Treatment of a platinum-refractory disease is discussed in appropriate guidelines [26] or reviewed elsewhere [8,18,27]. Importantly, whereas a study showed no superiority of a high-dose strategy compared to a conventional dose [28], it has been suggested that, at least in some tumors, there is actually only a partial resistance to platinum that may be overcome through high-dose chemotherapy [29]. In other words, part of the resistant disease could be a consequence of undertreatment. Multiple drug combinations are, indeed, available [18,26] that can be combined with surgery; in patients undergoing salvage treatment, around 40% of residual masses contain viable nonteratoma disease, and surgical excision should be pursued whenever technically possible independently of the size of the mass to improve the outcome [18]. “Desperation” (salvage) surgery can also be beneficial in certain patients with no more systemic options available, as opposed to palliative chemotherapy [30]. Late relapses (more than two years after completing systemic treatment), which are rare events (1–3% of patients with advanced disease), constitute another challenge; studying the biology of these tumors may provide insights on the emergence of cisplatin-resistant phenotype as well, since they seem to be enriched in yolk sac tumor morphology and include events of somatic (non-germ cell) malignancy and present with high microsatellite instability (MSI) and *BRAF* mutations [31]. Novel molecularly targeted therapies are currently under study, some in clinical trials, but have not yet produced results leading to integration in the clinic, probably due to the pathobiological heterogeneity of the disease and selection of patient cohorts [18]. This also indicates that cisplatin resistance should be multifactorial and that targeting a single marker will not be sufficient to reverse the phenotype, enhancing the need for establishing specific biomarkers of response to specific drugs.

## 3. Models for Studying Cisplatin Resistance Biology

**Figure 1:**
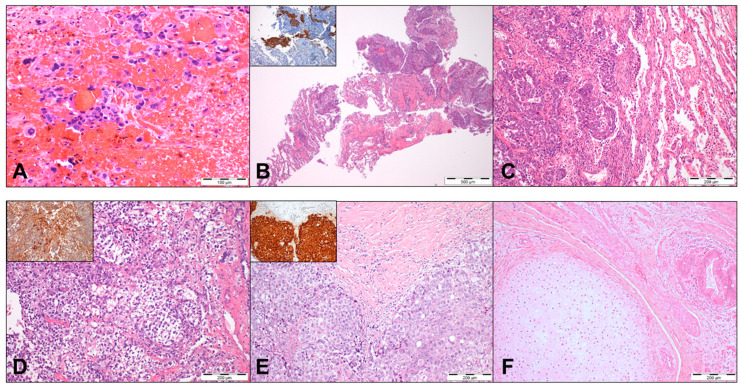
Illustrative histopathological examples of the infrequent tumor specimens from patients with the metastatic cisplatin-resistant disease. (**A**) A brain metastasis of a 35-year-old patient, presenting with stage III disease, undergoing multiple cisplatin-based courses of treatment, showing disease progression. The patient underwent excision of the brain metastasis, showing choriocarcinoma, in a bloody background. (**B**) A lung biopsy of a 21-year-old patient with a lung metastasis in the form of embryonal carcinoma, representing the disease progression after a first-line platinum treatment. Inset: tumor cells showed an immunoexpression of OCT3/4. (**C**) The previous patient was treated with multiple courses of cisplatin-based chemotherapy, but the disease progressed. He underwent salvage surgery, with a resection of lung metastases again showing a persistence of embryonal carcinoma, as illustrated in the figure, but ended up dying from disease. (**D**) A brain metastasis of a 25-year-old patient with stage III disease at presentation. He was treated with multiple courses of cisplatin-based chemotherapy, but the disease progressed under treatment with the emergence of a brain metastasis showing a yolk sac tumor histology. Inset: tumor cells showed a diffuse immunoexpression of alpha-fetoprotein. The patient underwent brain resection and radiotherapy to the brain but died from disease. (**E**,**F**) A post-chemotherapy retroperitoneal lymph-node dissection of a metastatic mass in a stage II patient. Dedicated sampling of the mass led to the finding of a small foci of a residual, viable nonteratoma disease, in the form of embryonal carcinoma, within a background of fibrosis and necrosis. (**E**) Inset: tumor cells showed an immunoexpression of OCT3/4. Within the specimen areas of the residual, mature teratoma were also present (**F**), with evidence of mature cartilage, smooth muscle, and intestinal epithelium. Cases were treated in our institute and retrieved from the pathology archive.

The challenge of studying cisplatin resistance biology is clear if one takes into consideration both the low frequency of the event and the lack of access to histological material from these patients (Figure 1). Accurate pathological assessment of the primary tumor by a GCT-dedicated pathologist is of utmost relevance for establishing prognosis and adjusting treatment strategies [5,25,32]. However, patients with known and previously treated metastatic disease that develop cisplatin resistance do not always undergo surgery or biopsy (either because the patient has no clinical conditions, it is not technically feasible, or it is risky – like in the case of brain metastases – or because it is simply deemed not necessary during the course of systemic treatment). This limits studies on cisplatin resistance biology in actual patient samples, with representation of the whole heterogeneity related to individual patient. Consensus guidelines for pathological handling of post-chemotherapy retroperitoneal lymph-node dissection (RPLND) specimens indicate the need for generous sampling (at least one block per centimeter of maximum diameter, although, very often, more sections should be made, making it very laborious), to clearly identify nonteratoma disease, which otherwise could be missed [33]. Indeed, subtypes such as seminoma are particularly sensitive to DNA-damaging agents, while others such as yolk sac tumor appear frequently in the cisplatin-resistant metastatic context, reflecting differences in biology. Overall, studies on cisplatin resistance making use of such chemo-exposed patient samples are scarce [31,34,35,36,37], and researchers often turn their attention to primary tumors of patients known to have developed resistance in the future, which is suboptimal given their chemo-naïve constitution [15]. Additionally, there is great heterogeneity within mixed tumors, with the cisplatin-resistant metastatic histological component not always being the dominant clone within the primary tumor; this is another argument in favor of interrogating the metastatic tumor instead of the primary. Indeed, the morphological heterogeneity is also accompanied by remarkable molecular differences among specific histological subtypes, as demonstrated in the integrated analyses of Shen et al. [38].

More commonly, research makes use of in vitro and in vivo preclinical models (Table 1). Indeed, many authors have studied cisplatin resistance mechanisms, phenotype, and the effects of specific drugs in the most well-characterized (T)GCT cell lines: NCCIT, 2102Ep, NTera-2 (non-seminoma-related cells) and TCam-2 (seminoma-like cell line) by making use of their matched cisplatin-resistant clones, obtained by culturing cells continuously and under long-term exposure to increasing doses of cisplatin (as described in detail elsewhere [20]). Importantly, dimethyl sulfoxide (DMSO), commonly used as a drug vehicle, was demonstrated to induce cisplatin resistance and differentiation in embryonal carcinoma cells, which should be taken into consideration when designing biological studies [39]. Moreover, in vivo mouse models of resistant disease can also be used by implanting cisplatin-resistant human tumor specimens or, more commonly, by injecting the resistant clones of (T)GCT cell lines available [40,41,42].

## 4. Dissecting Cisplatin Resistance Mechanisms

### 4.1. General Overview and Insights from Various Tumor Models

Cisplatin stands as one of the very first metal-based chemotherapy drugs, and it is widely used to treat cancer patients, including TGCTs but, also, head and neck, cervical, ovarian, bladder, and lung cancers [56]. It exerts its antineoplastic effects by acting in more than one front: binding to DNA and creating lesions (namely, protein-DNA complexes and inter/intra-strand DNA adducts, the latter being appointed as the most prominent lesion), which, eventually, are no longer amenable to be repaired by the existent DNA repair mechanisms; disrupting of the DNA, mRNA, and proteins’ syntheses; impeding replication; promoting the accumulation of reactive oxygen species; and activating signaling pathways that culminate in cell death [57]. Despite its clinical usefulness, two main factors currently limit the efficacy of cisplatin: drug-elicited toxicities (mainly dependent on the dosage), including nephrotoxicity, ototoxicity, neurotoxicity, hepatotoxicity, and gastrointestinal toxicity, among others (reviewed elsewhere [58]), and the emergence of resistance.

**Figure 2:**
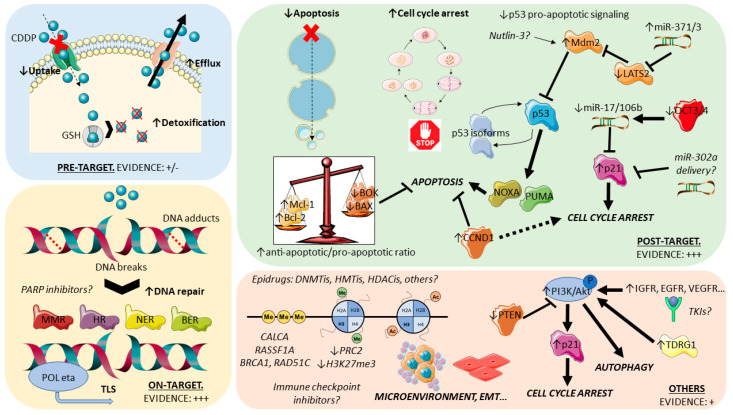
Pre-target, on-target, post-target, and off-target mechanisms involved in cisplatin resistance in (testicular) germ cell tumors. Most studies have focused on the on-target and post-target mechanisms (evidence: +++). Pre-target mechanisms are not thought to be major factors in the acquisition of cisplatin resistance in these tumors (evidence +/−). Other mechanisms are currently being explored, and related data is building up. A better understanding of these mechanisms underlies the development of specific effective therapies of cisplatin-resistant patients (italics). Notice that epigenetic (de)regulation can be found within any type of mechanism. See text for details. Abbreviations: TLS: translesion synthesis, EMT: epithelial-to-mesenchymal transition, and TKIs: tyrosine kinase inhibitors.

For organization purposes, cisplatin resistance has been organized by some authors [17,59,60,61] based in the order of events that follow introduction of the drug in the human body: pre-target mechanisms, on-target mechanisms, and post-target mechanisms (Figure 2). Pre-target mechanisms include those that occur before cisplatin actually binds to its targets in the cell; these are classical ways of (general) drug resistance and include decreased intracellular accumulation of the drug due to alterations in transporters and, also, increased efflux. Available studies have shown that efflux mechanisms are most likely not the main pre-target mechanism, with most evidence indicating reduced uptake as the main culprit. Within this mechanism, the involvement of copper transporters seems the most relevant, both in uptake and efflux (with the downregulation of *CTR1* and upregulation of *ATP7A/ATP7B* in cisplatin-resistant tumors, respectively) [62]. To date, however, available studies are scarce and show reduced expression of efflux pumps [63]. Pre-target mechanisms also comprise the upregulation of cytoplasmic scavenger players (like reduced glutathione—GSH [64]) that bind to cisplatin, hindering its action. Although some evidence pointing towards overall low levels of these detoxifier players in TGCTs (possibly contributing to the marked sensitivity to cisplatin) and upregulation in teratoma (cisplatin-resistant) [63,65], robust clinical evidence is lacking, with some studies reporting conflicting data [66,67]. Currently, pre-target mechanisms can be viewed as possibly contributive to resistance, but further work is needed focused on TGCTs and on patient material. On the other hand, on-target and post-target mechanisms have been the most studied (and, thus, will be further detailed in the following sections). The first refers to alterations that implicate DNA adducts formed upon cisplatin binding. Many studies have focused on the various DNA repair systems, as the cisplatin resistant phenotype is shown to gain the ability to repair DNA lesions more proficiently and faster [68] or, alternatively, is able to tolerate such lesions by the upregulation of specific polymerases that bypass DNA adducts [69]—both ways not triggering the desired apoptosis cascade, leading to tumor persistence. As for post-target mechanisms, they refer precisely to alterations in these final downstream signaling pathways that culminate in apoptosis. The disruption of signaling (including the p53/MDM2 axis) and of the apoptotic machinery itself (including factors like BCL2) have been implicated [70,71]. Lastly, several off-target mechanisms of resistance to this drug are being acknowledged that cause alterations in signaling cascades, which indirectly disrupt cisplatin actions and impedes cell death. These include, for instance, autophagy (generally activated in response to chemotherapy and being shown to be upregulated in cisplatin-resistant tumors [72]) or chaperones and receptors that exert pro-survival signals (such as heat shock protein 27—HSP27—and ERBB2 [73]). Evidence is building on the contributory role of these mechanisms in several cancer models that acquire a resistance to cisplatin and deserve explorations in studies dedicated to TGCTs. Indeed, a recent study on TCam-2 cell lines (including an in vivo mouse model) showed that testis developmental related gene 1 (*TDRG1*) promotes autophagy via the p110β/Rab5/Vps34 (PI3-kinase class III) cascade, contributing to cisplatin resistance [74]. Overall, and despite that specific studies for TGCTs are needed to confirm the relevance of these mechanisms, insights from other tumor models can be used as surrogates to pinpoint general mechanisms likely to be involved in cisplatin resistance (Table S1). The contribution of shifts in the epigenetic landscape of TGCTs has also been shown to impact the resistant phenotype. Additionally, specific pathways and cancer hallmarks were implicated in cisplatin resistance and deserve further exploration, such as the PI3K-AKT-mTOR (due to the frequent loss of *PTEN*) or IGF1R pathways (overactivated in resistant cells [75]).

In the following subsections, we will further dissect some of the mechanisms involved in cisplatin resistance that have been the focus of intense research, specifically in TGCTs.

### 4.2. TGCTs, Cisplatin Resistance, and DNA Damage Repair Systems

In a sense, the chemosensitivity of a specific neoplasm is dependent on its capacity to sense DNA damage and activate the so-called DNA damage response (DDR) and, importantly, on how it preferentially tends to respond to such damage (i.e., activating apoptosis or, on the contrary, cell cycle arrest and repair). In TGCTs, specifically, the expression levels of players involved in DNA repair is, overall, low (possibly relating to germ cell biology, programmed to follow an “all or nothing” fate decision and promote apoptosis to avoid transmitting DNA damage to the germline [68]); this is one of the reasons why these tumors are so sensitive to cisplatin, a DNA-damaging agent. It is not surprising, then, that an improved ability to repair DNA damage is a general feature commonly leading to cisplatin resistance.

The mismatch repair (MMR) DNA repair system, responsible for correcting small insertions or deletions, cannot, on its own, repair damage induced by cisplatin (i.e., the inter-strand adducts) but, importantly, is able to detect it (and, hence, activate apoptosis), especially *MLH1* and *MSH2* [59,76]. Hence, this concept of a “less-active MMR system, lower detection of errors, and lower signaling for apoptosis” has been explored in TGCTs as well: indeed, a lower expression of MMR proteins and related MSI have been associated to a cisplatin-resistant phenotype in clinical samples (and, additionally, *BRAF* mutations) [34,36]. MMR deficiency has long been recognized to occur in TGCTs and shown to affect prognosis [77], and, recently, a case attributed to a germline mutation (i.e., Lynch syndrome) has also been described, although this patient responded well to cisplatin treatment [78]. Some studies have pinpointed a correlation between tumor differentiation (OCT3/4 negativity, namely in teratomas, both chemo-naïve primaries, and chemo-treated metastases) and a relative decrease in MMR proteins expression, which would explain the less propensity for apoptosis in these subtypes [35,79,80]. However, some controversy in literature persists and more studies are needed to better explore the exact mechanism of how MMR proficiency interferes with cisplatin resistance. One study on 133 chemo-naïve primary TGCTs, 16% of which developed refractory disease, reported absence of *BRAF* mutations and of MSI [81]; however, studies including cisplatin-treated patient samples are more likely to tackle this matter [34]. Another mechanism worth exploring in the future is the one related to activation of translesion synthesis (TLS) induced by the absence of MMR proteins, which, in turn, potentiates skipping or bypassing of DNA adducts, again avoiding apoptotic-triggering [82]. Indeed, and as mentioned above, the increased action of specific polymerases (i.e., increased TLS) has also been connected to cisplatin resistance [69].

Theoretically, the nucleotide excision repair (NER) system should assume an important role in detecting and repairing cisplatin DNA adducts associated with distortion of the DNA helix. The low expression of players within this pathway (*XPA*, *XPF,* and *ERCC1*) in TGCTs has been implicated in extreme sensitivity to this drug [83,84]. The upregulation of *XPA* has been documented in resistant cell lines, and this has been translated to patient samples, where its expression additionally correlated with a poor prognosis and refractory disease [85]. *ERCC1* immunoexpression has also been suggested to associate with a lower sensitivity to cisplatin [86]. Additionally, interacting factors may affect the efficiency of this repair system; HMGB4 was shown to impede the function of the NER system in TGCTs, and in vitro knockout resulted in an increased resistance [87]. However, surprisingly, other studies similar in setting (both in vitro and using tissue samples) have failed to prove this shift in expression of NER proteins in resistant tumors [88,89,90]. More definitive data is needed to conclude on the role of NER in cisplatin sensitivity, but the implication of other DNA repair systems is also likely. For instance, and although the base excision repair (BER) system is responsible for DNA damage that does not distort the DNA helix, it could also be implicated in the sensitivity to ionizing radiation and bleomycin (often combined with cisplatin in the BEP regimen), as demonstrated by the increased in vitro resistance to this drug upon the overexpression of Ape1/ref1 [91]. However, the study also included a modest set of ten TGCT tissues all showing high expressions of this player across histologies, limiting the role of immunohistochemistry for discriminating sensitive and refractory cases.

Moreover, the homologous recombination (HR) DNA repair system, responsible for repairing double-strand breaks (which occur as a consequence of inter-strand crosslinks), is also limited in action in (T)GCT cell lines (with diminished RAD51 foci) [92]. This is particularly interesting given the advances in cancer treatment in tumor models that are defective in this pathway (taking advantage of synthetic lethality [93]), specifically with poly (ADP-ribose) polymerase (PARP) inhibitors; an in vitro study demonstrated the antitumor effect of this agent in monotherapy (dependent on the degree of HR deficiency and *PARP1* expression) and, additionally, potentiated its sensitivity to cisplatin [92]. PARP was shown to be overexpressed in TGCT tissues compared to normal testis [94]. The ongoing clinical trial with Olaparib in GCT patients (NCT02533765) will further elucidate on its clinical utility.

### 4.3. TGCTs, Cisplatin Resistance, the p53/Mdm2 Axis, and Apoptosis Initiation

A susceptibility to apoptosis is determinant for the intrinsic sensitivity of tumors to treatment [43]. The tumor suppressor p53 (so-called “guardian of the genome”) is activated upon DNA damage or abnormal cell growth and orchestrates the decision to enter cell-cycle arrest, either promoting cell repair or senescence or inducing apoptosis; these fate decisions are proportional to the severity and nature of the activating signal. Inactivation of p53 by mutations is common in somatic cancers, allowing cells with DNA damage to endure, promoting tumorigenesis [95]. The role of p53 in TGCTs is quite distinct [96,97]; since DDR is limited in these tumors, there is a low selective pressure for mutations in DDR players; hence, p53 mutations are hardly found in these tumors, which overexpress wild-type p53 [98,99]. They are known to have a “hyperactive p53 signaling” (meaning that, upon DNA damage, like treatments with cisplatin, the p53-orchestrated proapoptotic response precedes DNA repair) [100]. Embryonal carcinoma cells are hallmarked by functional p53, low levels of *MDM2* (which targets p53 for proteasomal degradation), and upregulation of OCT3/4, which induce members of the proapoptotic cascade *NOXA* and *PUMA* [101]. Studying the role of p53 in in vitro models can be of further help given the distinct p53 mutational status in representative cell lines (see above). It is certain that p53 mutations may emerge in cisplatin-resistant tumors [102,103], but other mechanisms of inactivation should be involved, contributing to resistance and poor outcome. The role of p53 in TGCTs and in cisplatin resistance is still controversial and may be very complex, since proapoptotic or antiapoptotic functions may develop in tumors, depending on the context (with p53 assuming a proapoptotic function in cisplatin-sensitive cells but protecting cells from apoptosis in intrinsically resistant cells) [104]. Some p53 family members (namely, p63 and p73) may further contribute to the equilibrium in case of p53 loss and may be epigenetically regulated, as demonstrated in studies of GTAp63 and Tap73 isoforms [105,106]. Additionally, it is known that miR-372 and miR-373, markers of these tumors (except teratoma; see below), downregulate the p53 pathway by targeting *LATS2* [107]. Moreover, there have been several reports that *MDM2* amplifications are enriched in cisplatin-resistant tumors by inactivating p53 and its proapoptotic program, which is supported by the efficacy of MDM2 inhibitor Nutlin-3 in cisplatin-resistant cases, promoting sensitivity to cisplatin [108,109,110]. Studies focusing solely on immunohistochemistry for p53 and *MDM2* in primary tumors have been conflicting, some failing to show ability to predict disease recurrence after cisplatin treatment and others showing up- and downregulations of MDM2/p53, respectively [70,111,112,113]. Current knowledge indicates the p53 pathway as a contributor to the cisplatin resistance phenotype but not solely responsible for cisplatin resistance. The role of other MDM2 family members, such as *MDM4*, could be explored in the future [114]. However, if, for TGCTs, evidence about the role of the p53 pathway is still debatable, it is building up in respect to mediastinal GCTs of nonseminoma histology. These have a distinct behavior, with a worse prognosis (accounted for in the IGCCCG risk classification), and studies aimed at dissecting their molecular pathobiology evidenced specificities that distinguish them from their gonadal counterparts [115,116]. In a recent study, Nappi et al. [115] have demonstrated that the amount of somatic mutations in mediastinal nonseminomas is higher compared to primary tumors and pointed out *TP53* mutations to determine poorer outcomes (with patients dying from disease), suggesting a more definitive role of p53 in these high-risk tumors, frequently being cisplatin-resistant. Confirmation of these findings in larger studies may lead to the emergence of targeted therapies for this aggressive form of the disease. Additionally, evidence is also building that leukemias, often associated with mediastinal nonseminoma GCTs, derive from a founding clone common to both entities, containing *TP53* and *PTEN* mutations, explaining the poor prognosis and resistance to treatment [117].

Moreover, further studies should be pursued on players directly involved in apoptotic machinery, as, for instance, the deregulation of the proapoptotic player *BOK*, which was associated with an enhanced resistance to cisplatin, possibly in equilibrium with the antiapoptotic *MCL1*, shown to be overexpressed in TGCTs [118,119]. While a high Bax/Bcl-2 ratio (i.e., high proapoptotic/antiapoptotic ratio) was identified to explain the hypersensitivity of GCT cells to etoposide [120], this was not confirmed regarding cisplatin resistance and not validated as a prognostic in metastatic patient samples [121,122]. Cell cycle regulation for sure can contribute to cisplatin resistance, as demonstrated by the differential (over)expression of *CCND1* (Cyclin D1) in platinum-resistant tumor samples [123]. Again, more definitive studies are lacking concerning these apoptosis/cell cycle effectors.

### 4.4. TGCTs, Cisplatin Resistance, and Epigenetics

Epigenetics is an important part of the tumorigenesis and classification model of TGCTs [3], and the generation of epigenetic-based biomarkers constitutes important progress in the field [124]. Targeting epigenetic alterations with specific epidrugs has been promising in the preclinical setting, despite some clinical studies showing limited benefits (see below). Nevertheless, there is evidence that DNA methylation profiles (namely, promoter hypermethylation of specific genes) is differentially distributed among cisplatin-sensitive and –resistant tumors [125]. Among the specific genes, *RASSF1A* promoter hypermethylation associates with cisplatin resistance, and molecular testing in liquid biopsies may be a reliable way of identifying these patients [126,127]. Moreover, *CALCA* hypermethylation associated with a cisplatin refractory disease [128]. The sensitivity to demethylating agents has been shown to correlate to high expression levels of DNA methyltransferase 3B (*DNMT3B*) [129,130]. The downstream mechanism is still debatable, but there is evidence relating to activation of p53 and other relevant targets being repressed by promoter methylation and, also, by altering pluripotency [131]. Indeed, a wide screening of the TCam-2 cell line after treatment with 5-azacytidine depicted interesting targets, such as demethylation of the tumor suppressor gene *KLF11* and the hypermethylation of *CFLAR*, which are advanced, to contribute to cisplatin resistance [132]. In the same way, a transcriptomics overview of distinct cisplatin-resistant cells showed a significant and shared enrichment on genes that, in normal instances, should be repressed by the polycomb repressive complex 2 (PRC2). Indeed, a decrease in PRC2 activity was demonstrated in resistant cells, accompanied by decreased H3K27me3 and expression of BMI1; moreover, validation of the findings was accomplished, as treatment with the *EZH2* inhibitor GSK-126 induced resistance in parental cells, while the opposite occurred upon treatment with histone lysine demethylase inhibitor GSK-J4 [133]. An integrated DNA methylation-expression study of pediatric intracranial nongerminomas also showed that miR-214-3p, which is regulated by promoter methylation, further contributes to cisplatin resistance by inhibiting Bcl2-like 11, a proapoptotic player [134]. Indeed, several microRNAs were shown to be differentially abundant among cisplatin-resistant cell lines (namely, the upregulation of miR-512/515/517/518/525 and downregulation of miR-99a/100/145). Given the interaction of the miR-371-373 cluster with p53, manipulating this axis may also prove useful in reverting cisplatin resistance [44], as could be the manipulation of miR-302a, induced upon treatment with cisplatin, reducing p21 levels and impeding G1 arrest [135].

Overall, it is key to understand epigenetic alterations leading to cisplatin resistance and, importantly, the mechanisms with which they interact, so that biomarkers can be generated and epidrugs can be combined with appropriate targeted treatments, achieving maximum antitumor effects. For instance, the hypermethylation of HR DNA repair genes *BRCA1* and *RAD51C* (see above) have been documented in 35% of NSs, and these may possibly be used as biomarkers for sensitivity to PARP inhibitors [38], like in other tumor models [136]. Another example is the epigenetic regulation of p53, namely by activity of the methyltransferases *SMYD2* and *PR-SET7*, which leads to inactivation of p53 in NTera-2 cell lines [137,138,139,140].

### 4.5. Seeking for Relevant Mutations/Copy Number Alterations Related to Cisplatin Resistance

Despite many research efforts, a remarkable and more definitive genetic feature (mutation or copy number alteration [CNA]) is still missing that generates the cisplatin-resistant phenotype. However, some works have retrieved relevant data from more “genome-wide” approaches. Two recent studies, making use of cell line models made resistant to cisplatin or cisplatin-resistant TGCTs (primaries and metastases, the largest series so far on whole genome sequencing), demonstrated that a resistant disease depicted significantly more CNAs (such as losses of chromosomes 1, 4, and 18 and gains of chromosome 8); single nucleotide polymorphisms (SNPs); and higher tumor mutational burdens (TMBs) [15,37]. One study found alterations in genes already known to be involved in the risk of acquiring TGCTs (*DMRTA1*), others on genes previously known to contribute to resistance (*MDM2,* although the association was not confirmed on the second mentioned study [37]), and uncovered novel targets (*ATRX* and *NSD1*) [15]. Both these new targets are of interest and reinforce the connection of cisplatin resistance with epigenetics (see above), as the former represents a chromatin remodeler (of the SWI/SNF family), and the latter constitutes a histone methyltransferase. Another work, including both primary and matched metastatic cisplatin refractory samples, was able to pinpoint mutations in genes, clonal in nature, that are potential effectors of cisplatin resistance; not surprisingly, again, these included mutations/amplifications in genes related to apoptosis (*RHBDD1*); DNA repair (*XRCC2*); p53 regulation (*MDM2*, also showed in another study using matched primary-metastatic samples [141]); and oncogenic signaling (*KIT*, *NRAS*, and *PIK3CA*) [142]. Authors highlight that mutations like the one on *XRCC2* (or in other HR DNA repair genes) are already present in the chemo-naïve primary TGCT, which is then selected by cisplatin treatment and becomes dominant in the refractory disease. Finally, recently, Necchi et al. [143] performed a wide study on 107 chemotherapy-exposed and relapsed TGCTs, demonstrating that *KRAS* alterations (mainly amplifications) are particularly common, possibly indicating that targeting the *KRAS* pathway may be effective in treating cisplatin-resistant tumors. Besides the higher TMB in relapsed nonseminomas, additional pathways with relevant alterations were the ones related to the cell cycle and *TP53*.

## 5. Differentiation-Dependent Cisplatin Resistance: Specificities of Teratoma

**Figure 3:**
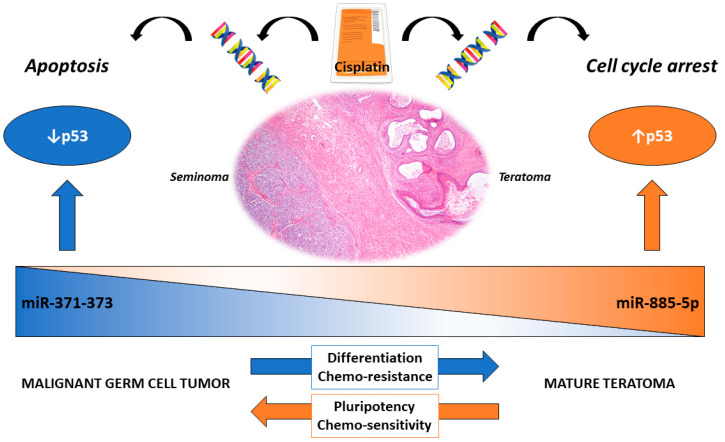
The proposed “microRNA switch” occurring in testicular germ cell tumors related to differentiation. MicroRNAs of the 371–373 cluster are upregulated in malignant subtypes (here represented by a seminoma histology) and target p53 (there are hardly any TP53 mutations in these tumors). The response to cisplatin is a proapoptotic response. In differentiated mature teratoma, on the contrary, this cluster is negative, and miR-885-5p is upregulated. The latter is a p53 activator. The response to cisplatin is directed towards cell cycle arrest, hence the clinical cisplatin resistance.

Within the histological subtypes of GCTs lies the most differentiated form of the disease, the teratoma. It is composed of mixtures of somatic-type tissues of the three germ layers (ectoderm, mesoderm, and endoderm) [144] and may arise, like other GCTs, in males and females, either in the gonads (testis and ovary) or in extragonadal locations. Within TGCTs, teratoma may be seen in two distinct settings according to the WHO 2016 classification: the prepubertal-type teratomas (actually not uncommon after pubertal age [5,145]), non-GCNIS-related, with benign behavior (which include the so-called epidermoid/dermoid cysts [146]), and the post-pubertal-type teratomas, frequently associated with other GCT components and commonly consisting of tissues with variable degrees of maturity, with malignant behaviors [147]. There are several reasons for also focusing on this subtype, specifically; despite being cytogenetically similar to the other GCT components, it is resistant to cisplatin treatment [148], as during differentiation, there is a growing disruption of the proapoptotic response to the drug, with the equilibrium shifting towards cell cycle arrest, repair, and quiescence instead [22,149]. Accordingly, teratoma was found to express *RB* and p21 and to show a low Bax/Bcl-2 ratio [61]. Furthermore, mature teratoma is among the most common subtypes emerging in post-chemotherapy masses, and the treatment approach differs in these patients (with surgical resection—which is technically challenging—being the available option [150]) compared to those with viable (nonteratoma) GCT, who can undergo salvage chemotherapy [25,151]. Interestingly, the most promising liquid biopsy-based biomarker of GCTs, the embryonic microRNA miR-371a-3p (for a broad review, refer to [152]), falls short on the detection of this subtype, specifically (due to the its differentiated nature). We have recently proposed a model of “microRNA switch”, where the upregulation of miR-371a-3p in nonteratomatous GCT targets *TP53*, while across differentiation towards teratoma, miR-371a-3p is replaced by miR-885-5p, which is upregulated and activates *TP53,* possibly contributing to the differential response to cisplatin [40] (Figure 3). The exact mechanism underlying this theory should be further explored. 

Overall, this differentiation-dependent cisplatin resistant phenotype most likely differs substantially in molecular landscape from the cisplatin resistance occurring in a nonteratoma (nondifferentiated) disease. Nevertheless, insights can be drawn from it. As a model to study the role of differentiation in resistance, most studies have been treating the several (T)GCT cell lines with all-trans retinoic acid (ATRA), inducing such differentiation [153,154,155,156]. Related to cisplatin resistance, ATRA was demonstrated to provoke the downregulation of *PUMA* and *NOXA*, abrogating apoptosis elicited by these factors [101]. Logically, differentiation is accompanied by the downregulation of the transcription factor *POU5F1*/OCT3/4 (reflecting the immunoexpression pattern of these neoplasms), which has also been linked to increased resistance to cisplatin [157], namely by downregulating the aforementioned *NOXA* and *PUMA* but, also, the miR-17/-106b cluster, potentiating the activation of p21 [51,101,158]. Taylor-Weiner et al. [157] indeed showed that both *NANOG* and *POU5F1* were not expressed in tumors obtained from cisplatin-resistant metastases, like for the in vivo mouse model of a sensitive and resistant disease, where the xenografts originating on cisplatin-resistant cell lines exhibited areas with cells of embryonal carcinoma morphology, but with the absence of OCT3/4 expression [53]. However, although OCT3/4 may have a contributory role on its own by downregulating pluripotency signatures [148], this was not fully supported by validation studies (with no difference in staining patterns between sensitive and resistant GCTs), and, also, because other subtypes than teratoma (choriocarcinoma and yolk sac tumor) are negative for this marker and still respond to cisplatin-based treatment [159]. Additionally, available trials with ATRA treatment have failed to produce clinical benefit in GCT patients [160]. Authors indeed recognize that the loss of pluripotency markers may be a driver of resistance but, also, simply be a consequence of changes in the methylation profile related to differentiation after exposure to chemotherapy (or a combination of both) [157]. More recently, Pierpoint et al. [161] developed a mouse model of metastatic TGCT with an expression of OCT3/4 that allowed them to study the effects of cisplatin on the EC cell population. They showed that a treatment with cisplatin selectively depleted these OCT3/4-positive cancer stem cells (by apoptosis), demonstrating in vivo why DNA-damaging agents are particularly effective in TGCTs compared to other somatic cancers.

Other particular aspects of teratoma are the growing teratoma syndrome (GTS) and teratomas with malignant somatic-type transformation. GTS is a potentially life-threatening condition that is clinically and therapeutically challenging [162]. It was recognized by Logothetis in 1982 [163], and it is clinically defined as a growing GCT after chemotherapy accompanied by a decrease of the classical serum tumor markers [164,165]. The biopathology of this phenomenon is still a matter of debate, but one theory is that embryonal carcinoma cells are induced to differentiate during treatment, assuming the form of teratoma-forming transit-amplifying cells. These cells endure the cisplatin-based treatment (as opposed to embryonal carcinoma cells, which die) and further differentiate into a teratoma, which continues to grow upon continuous treatment [164]. Study of this intriguing phenomenon may also shed light on cisplatin resistance related to differentiation. However, this is an infrequent occurrence, with most events figuring in the literature as case reports (refer to Tables S2–S4 for a brief overview of the most recent—2014–2019—descriptions of these events in the testis, ovary, and in extragonadal locations, presented chronologically), and larger multicenter cohorts would be beneficial for better understanding its biology and improving clinical care. Moreover, another rare phenomenon is the emergence of a somatic-type malignancy within teratoma, mostly in the form of sarcomas (50%) but, also, primitive neuroectodermal tumors (PNETs) or carcinomas. This occurs mostly in the metastatic setting, although, possibly, also in the primary tumor [166,167,168,169]. These patients show an aggravated prognosis compared to other GCT patients, and for this reason, treatments may differ. Most notably, these phenotypes are intrinsically highly resistant to cisplatin, with surgical excision being the only option, which should be always attempted [166]. Although signatures typical of GCTs are kept within these tumor forms, it is suggested that the overgrowth of immature elements and activation of specific oncogenes known to be relevant players in the specific types of somatic cancers (in their own native location) are behind the pathogenesis of the phenomenon [170]. There is a lack of large molecular studies on this topic (mostly individual reports [171]), which should be pursued in the future for a better understanding of this event, leading to better therapeutic alternatives to these patients.

## 6. Seeking for Novel Treatments Options for Cisplatin-Resistant Diseases

Uncovering new treatment agents for TGCT patients is challenging, given the overall lack of targetable alterations (such as mutations), like in other tumor types [172]. A summary of some of the recent drugs showing promise and being tested as targeted therapies for treating TGCT patients developing cisplatin resistance is presented in Table 2.

### 6.1. Immunotherapies

Given the recent advances in immunotherapies for cancer treatments, attention has been directed towards these anti-PD-1/anti-PD-L1 agents [173,174]. The immune microenvironment of TGCTs is particularly rich and distinct among subtypes [35,175], and the immunoexpression of immune checkpoints was demonstrated to have prognostic significance [176,177]. However, studies with these agents in cisplatin-relapsed patients with multiple-treatment courses were, so far, mainly nonbeneficial [178,179,180], except for isolated case reports [181] and, for some preliminary data, indicative of the clinical activity of durvalumab and tremelimumab [179]. One issue worth considering is that these studies were performed in multiple relapsed unselected populations; finding a reliable biomarker for discriminating the subset of patients that will benefit from such therapies may be the key for clinical success (like demonstrated in some reports, where patients with PD-L1-positive tumors benefited from the combination of anti-PD-1 pembrolizumab with standard chemotherapy [182]). Immunotherapies may be effective in the treatment of cisplatin-resistant tumors if the right setting is found [183].

### 6.2. Epidrugs

Also, given the role of epigenetics in TGCTs overall and influence in cisplatin resistance (discussed above), studies have also focused on epidrugs, alone or combined with other agents [16,21]. Demethylating agents (such as guadecitabine) have shown promising results in in vitro and pre-clinical in vivo studies, namely by resensitizing tumor cells to cisplatin [129,131,184,185,186]. Although more convincing data is lacking in clinical studies, which are not so remarkable thus far [187], data from ovarian GCT patients point towards clinical efficacy and provide data to proceed to phase II trials [188]. Other agents such as HDAC inhibitors [189,190], BET inhibitors [191], or, more recently, *ARID1A* inhibitors [192] have also shown good antitumor effect in vitro and should be explored in the future, possibly in combination with demethylating agents, as shown in a study of ovarian cancer cells exposed to decitabine and HDAC inhibitor belinostat [186]. A combination of epidrugs with immunotherapies could be envisaged as a good treatment alternative, given the known epigenetic regulation of the immune system [193]. Most likely, a multimodal approach, with platinum-based therapy combined with immunotherapies and epidrugs, will attain the greatest success by targeting and modulating the several mechanisms of cisplatin resistance, promoting maximum tumor death [16].

### 6.3. Others

Novel drugs are currently being explored as either alternatives or combination partners of cisplatin, some of them already being tested in clinical settings [42,194,195]. Follow-up studies on previous data can be envisioned, since novel therapeutic agents are becoming available; for instance, telomerase activity has been suggested to also relate to cisplatin efficacy (although data is controversial) and telomerase or CDK inhibitors could be explored [196,197,198]. This is a field of research with potential for expanding in the near future, with several clinical trials currently ongoing (for a recent review, refer to [21]).

## 7. Conclusions

Cisplatin resistance is one of the major challenges in the clinical approach to TGCT patients. The acquisition of resistance to platinum-based drugs is common to other tumor models, like bladder and ovarian cancer, all having in common a poor prognosis and lack of effective alternative therapies. The biopathology of this phenomenon is most likely multifactorial (although a major, still unidentified driver alteration is possible), with several concurrent mechanisms leading to the generation, selection, and propagation of a resistant clone. This way, combination treatment strategies that target multiple pathways probably will achieve greatest success, especially if novel delivery options are uncovered that reduce associated toxicities. Additional related challenges include the generation of biomarkers that predict the development of resistance and of those that, once resistance is recognized, predict the response to targeted treatments. The use of available and well-characterized cisplatin resistance preclinical models will continue to be instrumental for pointing towards the best treatment options. Moreover, liquid biopsies are expanding and gaining terrain in the TGCT field, especially with miR-371a-3p, proving itself as the most accurate noninvasive biomarker for diagnosis and follow-up of the disease, getting closer to integration in the clinic [152]. In the same way, liquid biopsy biomarkers predictive of resistance would be of great value in the field, since no other markers are available to fulfil this clinical need. The prospective collection of liquid biopsy samples from these pediatric and adult GCT patients developing a resistance to treatment, and their proper processing and storage, accompanied by the proper biobanking of tissue samples and international cooperation, will allow us to improve the clinical management and quality of life of TGCT patients.

## Figures and Tables

**Table 1:** In vitro and in vivo models for studying a cisplatin-resistant disease in (testicular) germ cell tumors.

In Vitro Models		
Cell Line	Features	Resistant Clone and References
NCCIT	Primary mediastinal. Adult male. Mixed nonseminoma (with embryonal carcinoma). p53-mutated [43].	NCCIT-R [42,44]
2102Ep	Primary testicular. Adult male. Mixed nonseminoma (with embryonal carcinoma). Wild-type p53 [43].	2102Ep-R [44,45]
NTera-2	Primary testicular. Adult male. Embryonal carcinoma. Wild-type p53 [43].	NT2-R [42,44]
TCam-2	Primary testicular. Adult male. Seminoma. *BRAF* V600E mutation [46,47].	TCam-2-R [48]
Others less commonly used	NOY-1-R (yolk sac tumor), H12.1RA, H12.1D, 1411HP, 1777NRpmet, GCT44 (mixed tumor), Tera-CP, 833K-R (embryonal carcinoma), GCT27-R, SuSa-R (teratoma), and P19 (mouse-derived and embryonal carcinoma).	[48,49,50,51,52,53,54,55]
**In vivo models**		
Injection of resistant cell lines.		[42,53]
Implantation of human tissue samples.		[41]

**Table 2:** Summary of some recent studies exploring novel treatment options for cisplatin-resistant testicular germ cell tumors.

Drug	Main Mechanism/Drug Class	Main Findings	Reference
Cabazitaxel	Chemotherapy; Taxane (microtubule inhibitor)	Clinical study. Limited activity in heavily treated refractory GCTs.	[195]
Dissulfiram	ALDH inhibitor (cancer stem cell marker)	Pre-clinical study. Synergistic antitumor effect (+ cisplatin) in an in vivo model. Restores sensitivity to cisplatin.	[42]
Everolimus	mTOR inhibitor	Clinical study. Limited activity in heavily treated refractory GCTs.	[194]
Sunitinib	RTK inhibitor (including VEGFR)	Pre-clinical study. Antitumor effect (including a reduced vasculature) in an in vivo model, including of a cisplatin resistant disease.	[199]
Avelumab	Immunotherapy; PD-L1 inhibitor	Clinical study. Well-tolerated but limited activity in heavily treated refractory GCTs.	[180]
Pembrolizumab	Immunotherapy; PD-1 inhibitor	Clinical study. Well-tolerated but limited activity in heavily treated refractory GCTs.	[178]
Durvalumab and Tremelimumab	Immunotherapy; anti-PD-L1 and anti-CTLA4	Clinical study. Partial response and stable disease in 2 patients; remaining with disease progression.	[179]
5-azacytidine	Epigenetics; Demethylating agent (nucleoside analog)	Pre-clinical study. Proapoptotic effect at low nM concentrations and overcomes cisplatin resistance.	[184]
Decitabine	Epigenetics; Demethylating agent (nucleoside analog)	Pre-clinical study. Causes DNA damage, promotes p53 and p21 activation, downregulates pluripotency factors, and activates *ATM*. Restores sensitivity to cisplatin.	[129,131]
Guadecitabine	Epigenetics; Demethylating agent (nucleoside analog)	Pre-clinical study. Restores sensitivity to cisplatin in in vivo models.	[185]
JQ1	Epigenetics; BET inhibitor	Pre-clinical study. Induces apoptosis, more prominent in resistant clones compared to parental.	[191]
C63 and BRD-K98645985	Epigenetics; *ARID1A* (chromatin remodeler) inhibitor	Pre-clinical study. *ARID1A* inhibitors sensitizes cisplatin-resistant cells to ATR inhibition.	[192]
Romidepsin	Epigenetics; HDAC inhibitor	Pre-clinical study. Antitumor effect, including in cisplatin-resistant cells.	[190]
Olaparib	DNA repair; PARP inhibitor	Pre-clinical study. Antitumor effect as monotherapy and sensitization to cisplatin.	[92]

Abbreviations: GCT: germ cell tumor, HDAC: histone deacetylase, mTOR: mammalian target of rapamycin, PARP: poly (ADP-ribose) polymerase, RTK: receptor tyrosine kinase, and VEGFR: vascular endothelial growth factor receptor.

## Supplementary Materials

The following are available online at https://www.mdpi.com/2072-6694/12/6/1601/s1. Table S1: Summary of some recent studies exploring cisplatin resistance in tumor models other than testicular germ cell tumors. Table S2: Review of the literature regarding the growing teratoma syndrome with a testicular origin. Table S3: Review of the literature regarding the growing teratoma syndrome with an ovarian origin. Table S4: Review of the literature regarding the growing teratoma syndrome with an extragonadal origin.

## References

[1] Znaor A., Skakkebaek N.E., Rajpert-De Meyts E., Laversanne M., Kulis T., Gurney J., Sarfati D., McGlynn K.A., Bray F. (2019). Testicular cancer incidence predictions in Europe 2010–2035: A rising burden despite population ageing. Int. J. Cancer.

[2] Fedorova V.A., Kadyrova R.A., Slita A.V., Muryleva A.A., Petrova P.R., Kovalskaya A.V., Lobov A.N., Zileeva Z.R., Tsypyshev D.O., Borisevich S.S. (2019). Antiviral activity of amides and carboxamides of quinolizidine alkaloid (-)-cytisine against human influenza virus A (H1N1) and parainfluenza virus type 3. Nat. Prod. Res..

[3] Oosterhuis J.W., Looijenga L.H.J. (2019). Human germ cell tumours from a developmental perspective. Nat. Rev. Cancer.

[4] Berney D.M., Looijenga L.H., Idrees M., Oosterhuis J.W., Rajpert-De Meyts E., Ulbright T.M., Skakkebaek N.E. (2016). Germ cell neoplasia in situ (GCNIS): Evolution of the current nomenclature for testicular pre-invasive germ cell malignancy. Histopathology.

[5] Lobo J., Costa A.L., Vilela-Salgueiro B., Rodrigues A., Guimaraes R., Cantante M., Lopes P., Antunes L., Jeronimo C., Henrique R. (2018). Testicular germ cell tumors: Revisiting a series in light of the new WHO classification and AJCC staging systems, focusing on challenges for pathologists. Hum. Pathol..

[6] Giuliano C.J., Freemantle S.J., Spinella M.J. (2006). Testicular Germ Cell Tumors: A Paradigm for the Successful Treatment of Solid Tumor Stem Cells. Curr. Cancer Ther. Rev..

[7] Moul J.W., Dodge R.K., Robertson J.E., Paulson D.F., Walther P.J. (1991). The impact of the “cisplatin era” of treatment on survival in testicular cancer. World J. Urol..

[8] Allen J.C., Kirschner A., Scarpato K.R., Morgans A.K. (2017). Current Management of Refractory Germ Cell Tumors and Future Directions. Curr. Oncol. Rep..

[9] Dieckmann K.P., Richter-Simonsen H., Kulejewski M., Ikogho R., Zecha H., Anheuser P., Pichlmeier U., Isbarn H. (2018). Testicular Germ-Cell Tumours: A Descriptive Analysis of Clinical Characteristics at First Presentation. Urol. Int..

[10] Lobo J., Stoop H., Gillis A.J.M., Looijenga L.H.J., Oosterhuis W. (2019). Interobserver Agreement in Vascular Invasion Scoring and the Added Value of Immunohistochemistry for Vascular Markers to Predict Disease Relapse in Stage I Testicular Nonseminomas. Am. J. Surg. Pathol..

[11] El Charif O., Mapes B., Trendowski M.R., Wheeler H.E., Wing C., Dinh P.C., Frisina R.D., Feldman D.R., Hamilton R.J., Vaughn D.J. (2019). Clinical and Genome-wide Analysis of Cisplatin-induced Tinnitus Implicates Novel Ototoxic Mechanisms. Clin. Cancer Res..

[12] Inoue Y., Nakamura T., Nakanishi H., Oishi M., Hongo F., Okihara K., Mizutani S., Kuroda J., Ukimura O. (2018). Therapy-related acute myeloid leukemia and myelodysplastic syndrome among refractory germ cell tumor patients. Int. J. Urol..

[13] Kvammen O., Myklebust T.A., Solberg A., Moller B., Klepp O.H., Fossa S.D., Tandstad T. (2016). Long-term Relative Survival after Diagnosis of Testicular Germ Cell Tumor. Cancer Epidemiol. Prev. Biomark..

[14] Bokemeyer C., Kollmannsberger C., Harstrick A., Beyer J., Gerl A., Casper J., Metzner B., Hartmann J.T., Schmoll H.J., Kanz L. (1999). Treatment of patients with cisplatin-refractory testicular germ-cell cancer. German Testicular Cancer Study Group (GTCSG). Int. J. Cancer.

[15] Bakardjieva-Mihaylova V., Skvarova Kramarzova K., Slamova M., Svaton M., Rejlova K., Zaliova M., Dobiasova A., Fiser K., Stuchly J., Grega M. (2019). Molecular Basis of Cisplatin Resistance in Testicular Germ Cell Tumors. Cancers (Basel).

[16] Oing C., Skowron M.A., Bokemeyer C., Nettersheim D. (2019). Epigenetic treatment combinations to effectively target cisplatin-resistant germ cell tumors: Past, present, and future considerations. Andrology.

[17] Singh R., Fazal Z., Freemantle S.J., Spinella M.J. (2019). Mechanisms of cisplatin sensitivity and resistance in testicular germ cell tumors. Cancer Drug Resist..

[18] Oing C., Seidel C., Bokemeyer C. (2018). Therapeutic approaches for refractory germ cell cancer. Expert Rev. Anticancer Ther..

[19] Kalavska K., Conteduca V., De Giorgi U., Mego M. (2018). Molecular Mechanisms of Resistance in Testicular Germ Cell Tumors-clinical Implications. Curr. Cancer Drug Targets.

[20] Schmidtova S., Kalavska K., Kucerova L. (2018). Molecular Mechanisms of Cisplatin Chemoresistance and Its Circumventing in Testicular Germ Cell Tumors. Curr. Oncol. Rep..

[21] Oing C., Alsdorf W.H., von Amsberg G., Oechsle K., Bokemeyer C. (2017). Platinum-refractory germ cell tumors: An update on current treatment options and developments. World J. Urol..

[22] Jacobsen C., Honecker F. (2015). Cisplatin resistance in germ cell tumours: Models and mechanisms. Andrology.

[23] Koster R., van Vugt M.A., Timmer-Bosscha H., Gietema J.A., de Jong S. (2013). Unravelling mechanisms of cisplatin sensitivity and resistance in testicular cancer. Expert Rev. Mol. Med..

[24] Lorch A., Beyer J., Bascoul-Mollevi C., Kramar A., Einhorn L.H., Necchi A., Massard C., De Giorgi U., Flechon A., International Prognostic Factors Study Group (2010). Prognostic factors in patients with metastatic germ cell tumors who experienced treatment failure with cisplatin-based first-line chemotherapy. J. Clin. Oncol..

[25] Verrill C., Yilmaz A., Srigley J.R., Amin M.B., Comperat E., Egevad L., Ulbright T.M., Tickoo S.K., Berney D.M., Epstein J.I. (2017). Reporting and Staging of Testicular Germ Cell Tumors: The International Society of Urological Pathology (ISUP) Testicular Cancer Consultation Conference Recommendations. Am. J. Surg. Pathol..

[26] Honecker F., Aparicio J., Berney D., Beyer J., Bokemeyer C., Cathomas R., Clarke N., Cohn-Cedermark G., Daugaard G., Dieckmann K.P. (2018). ESMO Consensus Conference on testicular germ cell cancer: Diagnosis, treatment and follow-up. Ann. Oncol..

[27] Koychev D., Oechsle K., Bokemeyer C., Honecker F. (2011). Treatment of patients with relapsed and/or cisplatin-refractory metastatic germ cell tumours: An update. Int. J. Androl..

[28] Pico J.L., Rosti G., Kramar A., Wandt H., Koza V., Salvioni R., Theodore C., Lelli G., Siegert W., Horwich A. (2005). A randomised trial of high-dose chemotherapy in the salvage treatment of patients failing first-line platinum chemotherapy for advanced germ cell tumours. Ann. Oncol..

[29] Lorch A., Bascoul-Mollevi C., Kramar A., Einhorn L., Necchi A., Massard C., De Giorgi U., Flechon A., Margolin K., Lotz J.P. (2011). Conventional-dose versus high-dose chemotherapy as first salvage treatment in male patients with metastatic germ cell tumors: Evidence from a large international database. J. Clin. Oncol..

[30] Albers P., Ganz A., Hannig E., Miersch W.D., Muller S.C. (2000). Salvage surgery of chemorefractory germ cell tumors with elevated tumor markers. J. Urol..

[31] Mayer F., Wermann H., Albers P., Stoop H., Gillis A.J., Hartmann J.T., Bokemeyer C.C., Oosterhuis J.W., Looijenga L.H., Honecker F. (2011). Histopathological and molecular features of late relapses in non-seminomas. BJU Int..

[32] Ronchi A., Pagliuca F., Franco R. (2019). Testicular germ cell tumors: The changing role of the pathologist. Ann. Transl. Med..

[33] Verrill C., Perry-Keene J., Srigley J.R., Zhou M., Humphrey P.A., Lopez-Beltran A., Egevad L., Ulbright T.M., Tickoo S.K., Epstein J.I. (2018). Intraoperative Consultation and Macroscopic Handling: The International Society of Urological Pathology (ISUP) Testicular Cancer Consultation Conference Recommendations. Am. J. Surg. Pathol..

[34] Honecker F., Wermann H., Mayer F., Gillis A.J., Stoop H., van Gurp R.J., Oechsle K., Steyerberg E., Hartmann J.T., Dinjens W.N. (2009). Microsatellite instability, mismatch repair deficiency, and BRAF mutation in treatment-resistant germ cell tumors. J. Clin. Oncol..

[35] Lobo J., Rodrigues A., Guimaraes R., Cantante M., Lopes P., Mauricio J., Oliveira J., Jeronimo C., Henrique R. (2019). Detailed Characterization of Immune Cell Infiltrate and Expression of Immune Checkpoint Molecules PD-L1/CTLA-4 and MMR Proteins in Testicular Germ Cell Tumors Disclose Novel Disease Biomarkers. Cancers (Basel).

[36] Mayer F., Gillis A.J., Dinjens W., Oosterhuis J.W., Bokemeyer C., Looijenga L.H. (2002). Microsatellite instability of germ cell tumors is associated with resistance to systemic treatment. Cancer Res..

[37] Loveday C., Litchfield K., Proszek P.Z., Cornish A.J., Santo F., Levy M., Macintyre G., Holryod A., Broderick P., Dudakia D. (2020). Genomic landscape of platinum resistant and sensitive testicular cancers. Nat. Commun..

[38] Shen H., Shih J., Hollern D.P., Wang L., Bowlby R., Tickoo S.K., Thorsson V., Mungall A.J., Newton Y., Hegde A.M. (2018). Integrated Molecular Characterization of Testicular Germ Cell Tumors. Cell Rep..

[39] Kita H., Okamoto K., Kushima R., Kawauchi A., Chano T. (2015). Dimethyl sulfoxide induces chemotherapeutic resistance in the treatment of testicular embryonal carcinomas. Oncol. Lett..

[40] Lobo J., Gillis A.J.M., van den Berg A., Dorssers L.C.J., Belge G., Dieckmann K.P., Roest H.P., van der Laan L.J.W., Gietema J., Hamilton R.J. (2019). Identification and Validation Model for Informative Liquid Biopsy-Based microRNA Biomarkers: Insights from Germ Cell Tumor In Vitro, In Vivo and Patient-Derived Data. Cells.

[41] Piulats J.M., Vidal A., Garcia-Rodriguez F.J., Munoz C., Nadal M., Moutinho C., Martinez-Iniesta M., Mora J., Figueras A., Guino E. (2018). Orthoxenografts of Testicular Germ Cell Tumors Demonstrate Genomic Changes Associated with Cisplatin Resistance and Identify PDMP as a Resensitizing Agent. Clin. Cancer Res..

[42] Schmidtova S., Kalavska K., Gercakova K., Cierna Z., Miklikova S., Smolkova B., Buocikova V., Miskovska V., Durinikova E., Burikova M. (2019). Disulfiram Overcomes Cisplatin Resistance in Human Embryonal Carcinoma Cells. Cancers (Basel).

[43] Burger H., Nooter K., Boersma A.W., van Wingerden K.E., Looijenga L.H., Jochemsen A.G., Stoter G. (1999). Distinct p53-independent apoptotic cell death signalling pathways in testicular germ cell tumour cell lines. Int. J. Cancer.

[44] Port M., Glaesener S., Ruf C., Riecke A., Bokemeyer C., Meineke V., Honecker F., Abend M. (2011). Micro-RNA expression in cisplatin resistant germ cell tumor cell lines. Mol. Cancer.

[45] Nitzsche B., Gloesenkamp C., Schrader M., Hoffmann B., Zengerling F., Balabanov S., Honecker F., Hopfner M. (2012). Anti-tumour activity of two novel compounds in cisplatin-resistant testicular germ cell cancer. Br. J. Cancer.

[46] De Jong J., Stoop H., Gillis A.J., Hersmus R., van Gurp R.J., van de Geijn G.J., van Drunen E., Beverloo H.B., Schneider D.T., Sherlock J.K. (2008). Further characterization of the first seminoma cell line TCam-2. Genes Chromosomes Cancer.

[47] Mizuno Y., Gotoh A., Kamidono S., Kitazawa S. (1993). Establishment and characterization of a new human testicular germ cell tumor cell line (TCam-2). Nihon Hinyokika Gakkai Zasshi.

[48] Timmerman D.M., Lobo J., Gillis A., Remmers T., Schmidtova S., Kalavska K., Hulleman E., Oing C., Dorssers L., Ulbright T. (2020). Sequential 12q- and 3p-amplification is associated with cisplatin resistance in male germ cell cancers.

[49] Schaffrath J., Schmoll H.J., Voigt W., Muller L.P., Muller-Tidow C., Mueller T. (2017). Efficacy of targeted drugs in germ cell cancer cell lines with differential cisplatin sensitivity. PLoS ONE.

[50] Timmer-Bosscha H., Timmer A., Meijer C., de Vries E.G., de Jong B., Oosterhuis J.W., Mulder N.H. (1993). cis-Diamminedichloroplatinum(ii) resistance in vitro and in vivo in human embryonal carcinoma cells. Cancer Res..

[51] Mueller T., Mueller L.P., Luetzkendorf J., Voigt W., Simon H., Schmoll H.J. (2006). Loss of Oct-3/4 expression in embryonal carcinoma cells is associated with induction of cisplatin resistance. Tumor Biol..

[52] Perry J., Powles T., Shamash J., Veerupillai A., McGrowder E., Noel E., Lu Y.J., Oliver T., Joel S. (2009). The relative activity of cisplatin, oxaliplatin and satraplatin in testicular germ cell tumour sensitive and resistant cell lines. Cancer Chemother. Pharmacol..

[53] Mueller T., Mueller L.P., Holzhausen H.J., Witthuhn R., Albers P., Schmoll H.J. (2010). Histological evidence for the existence of germ cell tumor cells showing embryonal carcinoma morphology but lacking OCT4 expression and cisplatin sensitivity. Histochem. Cell Biol..

[54] Shibata K., Umezu T., Sakurai M., Kajiyama H., Yamamoto E., Ino K., Nawa A., Kikkawa F. (2011). Establishment of cisplatin-resistant ovarian yolk sac tumor cells and investigation of the mechanism of cisplatin resistance using this cell line. Gynecol. Obstet. Invest..

[55] Minucci S., Horn V., Bhattacharyya N., Russanova V., Ogryzko V.V., Gabriele L., Howard B.H., Ozato K. (1997). A histone deacetylase inhibitor potentiates retinoid receptor action in embryonal carcinoma cells. Proc. Natl. Acad. Sci. USA.

[56] Brown A., Kumar S., Tchounwou P.B. (2019). Cisplatin-Based Chemotherapy of Human Cancers. J. Cancer Sci. Ther..

[57] Galluzzi L., Vitale I., Michels J., Brenner C., Szabadkai G., Harel-Bellan A., Castedo M., Kroemer G. (2014). Systems biology of cisplatin resistance: Past, present and future. Cell Death Dis..

[58] Oun R., Moussa Y.E., Wheate N.J. (2018). The side effects of platinum-based chemotherapy drugs: A review for chemists. Dalton Trans..

[59] Galluzzi L., Senovilla L., Vitale I., Michels J., Martins I., Kepp O., Castedo M., Kroemer G. (2012). Molecular mechanisms of cisplatin resistance. Oncogene.

[60] Chen S.H., Chang J.Y. (2019). New Insights into Mechanisms of Cisplatin Resistance: From Tumor Cell to Microenvironment. Int. J. Mol. Sci..

[61] Mayer F., Honecker F., Looijenga L.H., Bokemeyer C. (2003). Towards an understanding of the biological basis of response to cisplatin-based chemotherapy in germ-cell tumors. Ann. Oncol..

[62] Ishida S., Lee J., Thiele D.J., Herskowitz I. (2002). Uptake of the anticancer drug cisplatin mediated by the copper transporter Ctr1 in yeast and mammals. Proc. Natl. Acad. Sci. USA.

[63] Mayer F., Stoop H., Scheffer G.L., Scheper R., Oosterhuis J.W., Looijenga L.H., Bokemeyer C. (2003). Molecular determinants of treatment response in human germ cell tumors. Clin. Cancer Res..

[64] Chen H.H., Kuo M.T. (2010). Role of glutathione in the regulation of Cisplatin resistance in cancer chemotherapy. Met. Drugs.

[65] Masters J.R., Thomas R., Hall A.G., Hogarth L., Matheson E.C., Cattan A.R., Lohrer H. (1996). Sensitivity of testis tumour cells to chemotherapeutic drugs: Role of detoxifying pathways. Eur. J. Cancer.

[66] Koropatnick J., Kloth D.M., Kadhim S., Chin J.L., Cherian M.G. (1995). Metallothionein expression and resistance to cisplatin in a human germ cell tumor cell line. J. Pharmacol. Exp. Ther..

[67] Meijer C., Timmer A., De Vries E.G., Groten J.P., Knol A., Zwart N., Dam W.A., Sleijfer D.T., Mulder N.H. (2000). Role of metallothionein in cisplatin sensitivity of germ-cell tumours. Int. J. Cancer.

[68] Bloom J.C., Loehr A.R., Schimenti J.C., Weiss R.S. (2019). Germline genome protection: Implications for gamete quality and germ cell tumorigenesis. Andrology.

[69] Srivastava A.K., Han C., Zhao R., Cui T., Dai Y., Mao C., Zhao W., Zhang X., Yu J., Wang Q.E. (2015). Enhanced expression of DNA polymerase eta contributes to cisplatin resistance of ovarian cancer stem cells. Proc. Natl. Acad. Sci. USA.

[70] Kersemaekers A.M., Mayer F., Molier M., van Weeren P.C., Oosterhuis J.W., Bokemeyer C., Looijenga L.H. (2002). Role of P53 and MDM2 in treatment response of human germ cell tumors. J. Clin. Oncol..

[71] Michaud W.A., Nichols A.C., Mroz E.A., Faquin W.C., Clark J.R., Begum S., Westra W.H., Wada H., Busse P.M., Ellisen L.W. (2009). Bcl-2 blocks cisplatin-induced apoptosis and predicts poor outcome following chemoradiation treatment in advanced oropharyngeal squamous cell carcinoma. Clin. Cancer Res..

[72] Ren J.H., He W.S., Nong L., Zhu Q.Y., Hu K., Zhang R.G., Huang L.L., Zhu F., Wu G. (2010). Acquired cisplatin resistance in human lung adenocarcinoma cells is associated with enhanced autophagy. Cancer Biother. Radiopharm..

[73] Zhang Y., Shen X. (2007). Heat shock protein 27 protects L929 cells from cisplatin-induced apoptosis by enhancing Akt activation and abating suppression of thioredoxin reductase activity. Clin. Cancer Res..

[74] Peng D., Wei J., Gan Y., Yang J., Jiang X., Kitazawa R., Xiang Y., Dai Y., Tang Y. (2019). Testis developmental related gene 1 regulates the chemosensitivity of seminoma TCam-2 cells to cisplatin via autophagy. J. Cell Mol. Med..

[75] Juliachs M., Munoz C., Moutinho C.A., Vidal A., Condom E., Esteller M., Graupera M., Casanovas O., Germa J.R., Villanueva A. (2014). The PDGFRbeta-AKT pathway contributes to CDDP-acquired resistance in testicular germ cell tumors. Clin. Cancer Res..

[76] Vaisman A., Varchenko M., Umar A., Kunkel T.A., Risinger J.I., Barrett J.C., Hamilton T.C., Chaney S.G. (1998). The role of hMLH1, hMSH3, and hMSH6 defects in cisplatin and oxaliplatin resistance: Correlation with replicative bypass of platinum-DNA adducts. Cancer Res..

[77] Olasz J., Mandoky L., Geczi L., Bodrogi I., Csuka O., Bak M. (2005). Influence of hMLH1 methylation, mismatch repair deficiency and microsatellite instability on chemoresistance of testicular germ-cell tumors. Anticancer Res..

[78] Lobo J., Pinto C., Pinheiro M., Lobo F., Sousa N., Lopes P., Looijenga L.H., Jeronimo C., Teixeira M.R., Henrique R. (2020). Widening the spectrum of Lynch syndrome: First report of testicular seminoma attributable to MSH2 loss. Histopathology.

[79] Rudolph C., Melau C., Nielsen J.E., Vile Jensen K., Liu D., Pena-Diaz J., Rajpert-De Meyts E., Rasmussen L.J., Jorgensen A. (2017). Involvement of the DNA mismatch repair system in cisplatin sensitivity of testicular germ cell tumours. Cell Oncol..

[80] Velasco A., Riquelme E., Schultz M., Wistuba I.I., Villarroel L., Pizarro J., Berlin A., Ittmann M., Koh M.S., Leach F.S. (2004). Mismatch repair gene expression and genetic instability in testicular germ cell tumor. Cancer Biol. Ther..

[81] Carcano F.M., Lengert A.H., Vidal D.O., Scapulatempo Neto C., Queiroz L., Marques H., Baltazar F., Berardinelli G.N., Martinelli C.M., da Silva E.C. (2016). Absence of microsatellite instability and BRAF (V600E) mutation in testicular germ cell tumors. Andrology.

[82] Bassett E., Vaisman A., Tropea K.A., McCall C.M., Masutani C., Hanaoka F., Chaney S.G. (2002). Frameshifts and deletions during in vitro translesion synthesis past Pt-DNA adducts by DNA polymerases beta and eta. DNA Repair.

[83] Welsh C., Day R., McGurk C., Masters J.R., Wood R.D., Koberle B. (2004). Reduced levels of XPA, ERCC1 and XPF DNA repair proteins in testis tumor cell lines. Int. J. Cancer.

[84] Koberle B., Masters J.R., Hartley J.A., Wood R.D. (1999). Defective repair of cisplatin-induced DNA damage caused by reduced XPA protein in testicular germ cell tumours. Curr. Biol..

[85] Cierna Z., Miskovska V., Roska J., Jurkovicova D., Pulzova L.B., Sestakova Z., Hurbanova L., Machalekova K., Chovanec M., Rejlekova K. (2020). Increased levels of XPA might be the basis of cisplatin resistance in germ cell tumours. BMC Cancer.

[86] Mendoza J., Martinez J., Hernandez C., Perez-Montiel D., Castro C., Fabian-Morales E., Santibanez M., Gonzalez-Barrios R., Diaz-Chavez J., Andonegui M.A. (2013). Association between ERCC1 and XPA expression and polymorphisms and the response to cisplatin in testicular germ cell tumours. Br. J. Cancer.

[87] Awuah S.G., Riddell I.A., Lippard S.J. (2017). Repair shielding of platinum-DNA lesions in testicular germ cell tumors by high-mobility group box protein 4 imparts cisplatin hypersensitivity. Proc. Natl. Acad. Sci. USA.

[88] Koberle B., Roginskaya V., Zima K.S., Masters J.R., Wood R.D. (2008). Elevation of XPA protein level in testis tumor cells without increasing resistance to cisplatin or UV radiation. Mol. Carcinog..

[89] Honecker F., Mayer F., Stoop H., Oosterhuis J.W., Koch S., Bokemeyer C., Looijenga L.H. (2003). Xeroderma pigmentosum group a protein and chemotherapy resistance in human germ cell tumors. Lab. Investig..

[90] Koberle B., Payne J., Grimaldi K.A., Hartley J.A., Masters J.R. (1996). DNA repair in cisplatin-sensitive and resistant human cell lines measured in specific genes by quantitative polymerase chain reaction. Biochem. Pharmacol..

[91] Robertson K.A., Bullock H.A., Xu Y., Tritt R., Zimmerman E., Ulbright T.M., Foster R.S., Einhorn L.H., Kelley M.R. (2001). Altered expression of Ape1/ref-1 in germ cell tumors and overexpression in NT2 cells confers resistance to bleomycin and radiation. Cancer Res..

[92] Cavallo F., Graziani G., Antinozzi C., Feldman D.R., Houldsworth J., Bosl G.J., Chaganti R.S., Moynahan M.E., Jasin M., Barchi M. (2012). Reduced proficiency in homologous recombination underlies the high sensitivity of embryonal carcinoma testicular germ cell tumors to Cisplatin and poly (adp-ribose) polymerase inhibition. PLoS ONE.

[93] Lord C.J., Ashworth A. (2017). PARP inhibitors: Synthetic lethality in the clinic. Science.

[94] Mego M., Cierna Z., Svetlovska D., Macak D., Machalekova K., Miskovska V., Chovanec M., Usakova V., Obertova J., Babal P. (2013). PARP expression in germ cell tumours. J. Clin. Pathol..

[95] Mantovani F., Collavin L., Del Sal G. (2019). Mutant p53 as a guardian of the cancer cell. Cell Death Differ..

[96] Cavallo F., Feldman D.R., Barchi M. (2013). Revisiting DNA damage repair, p53-mediated apoptosis and cisplatin sensitivity in germ cell tumors. Int. J. Dev. Biol..

[97] Riou G., Barrois M., Prost S., Terrier M.J., Theodore C., Levine A.J. (1995). The p53 and mdm-2 genes in human testicular germ-cell tumors. Mol. Carcinog..

[98] Bartkova J., Rajpert-De Meyts E., Skakkebaek N.E., Lukas J., Bartek J. (2007). DNA damage response in human testes and testicular germ cell tumours: Biology and implications for therapy. Int. J. Androl..

[99] Guillou L., Estreicher A., Chaubert P., Hurlimann J., Kurt A.M., Metthez G., Iggo R., Gray A.C., Jichlinski P., Leisinger H.J. (1996). Germ cell tumors of the testis overexpress wild-type p53. Am. J. Pathol..

[100] Gutekunst M., Oren M., Weilbacher A., Dengler M.A., Markwardt C., Thomale J., Aulitzky W.E., van der Kuip H. (2011). p53 hypersensitivity is the predominant mechanism of the unique responsiveness of testicular germ cell tumor (TGCT) cells to cisplatin. PLoS ONE.

[101] Gutekunst M., Mueller T., Weilbacher A., Dengler M.A., Bedke J., Kruck S., Oren M., Aulitzky W.E., van der Kuip H. (2013). Cisplatin hypersensitivity of testicular germ cell tumors is determined by high constitutive Noxa levels mediated by Oct-4. Cancer Res..

[102] Houldsworth J., Xiao H., Murty V.V., Chen W., Ray B., Reuter V.E., Bosl G.J., Chaganti R.S. (1998). Human male germ cell tumor resistance to cisplatin is linked to TP53 gene mutation. Oncogene.

[103] Bagrodia A., Lee B.H., Lee W., Cha E.K., Sfakianos J.P., Iyer G., Pietzak E.J., Gao S.P., Zabor E.C., Ostrovnaya I. (2016). Genetic Determinants of Cisplatin Resistance in Patients With Advanced Germ Cell Tumors. J. Clin. Oncol..

[104] Di Pietro A., Koster R., Boersma-van Eck W., Dam W.A., Mulder N.H., Gietema J.A., de Vries E.G., de Jong S. (2012). Pro- and anti-apoptotic effects of p53 in cisplatin-treated human testicular cancer are cell context-dependent. Cell Cycle.

[105] Grande L., Bretones G., Rosa-Garrido M., Garrido-Martin E.M., Hernandez T., Fraile S., Botella L., de Alava E., Vidal A., Garcia del Muro X. (2012). Transcription factors Sp1 and p73 control the expression of the proapoptotic protein NOXA in the response of testicular embryonal carcinoma cells to cisplatin. J. Biol. Chem..

[106] Beyer U., Moll-Rocek J., Moll U.M., Dobbelstein M. (2011). Endogenous retrovirus drives hitherto unknown proapoptotic p63 isoforms in the male germ line of humans and great apes. Proc. Natl. Acad. Sci. USA.

[107] Voorhoeve P.M., le Sage C., Schrier M., Gillis A.J., Stoop H., Nagel R., Liu Y.P., van Duijse J., Drost J., Griekspoor A. (2006). A genetic screen implicates miRNA-372 and miRNA-373 as oncogenes in testicular germ cell tumors. Cell.

[108] Bauer S., Muhlenberg T., Leahy M., Hoiczyk M., Gauler T., Schuler M., Looijenga L. (2010). Therapeutic potential of Mdm2 inhibition in malignant germ cell tumours. Eur. Urol..

[109] Koster R., Timmer-Bosscha H., Bischoff R., Gietema J.A., de Jong S. (2011). Disruption of the MDM2-p53 interaction strongly potentiates p53-dependent apoptosis in cisplatin-resistant human testicular carcinoma cells via the Fas/FasL pathway. Cell Death Dis..

[110] Li B., Cheng Q., Li Z., Chen J. (2010). p53 inactivation by MDM2 and MDMX negative feedback loops in testicular germ cell tumors. Cell Cycle.

[111] Garcia-Velasco A., Duran I., Garcia E., Taron M., Ballestin C., Castellanos D., Cortes-Funes H., Paz-Ares L. (2012). Biological markers of cisplatin resistance in advanced testicular germ cell tumours. Clin. Transl. Oncol..

[112] Mandoky L., Szende B., Geczi L., Bodrogi I., Kasler M., Bak M. (2008). Apoptosis regulation and spontaneous apoptosis index of testicular germ cell tumors are associated with differentiation and resistance to systemic treatment. Anticancer Res..

[113] Lobo J., Alzamora M.A., Guimaraes R., Cantante M., Lopes P., Braga I., Mauricio J., Jeronimo C., Henrique R. (2020). p53 and MDM2 expression in primary and metastatic testicular germ cell tumors: Association with clinical outcome. Andrology.

[114] Haupt S., Mejia-Hernandez J.O., Vijayakumaran R., Keam S.P., Haupt Y. (2019). The long and the short of it: The MDM4 tail so far. J. Mol. Cell Biol..

[115] Nappi L., Annala M., Vandekerkhove G., Fazli L., Gleave M., Chi K.N., Kollmannsberger C.K., Wyatt A.W. (2017). Molecular dissection of primary mediastinal germ cell tumors. J. Clin. Oncol..

[116] Ronchi A., Cozzolino I., Montella M., Panarese I., Zito Marino F., Rossetti S., Chieffi P., Accardo M., Facchini G., Franco R. (2019). Extragonadal germ cell tumors: Not just a matter of location. A review about clinical, molecular and pathological features. Cancer Med..

[117] Lu C., Riedell P., Miller C.A., Hagemann I.S., Westervelt P., Ozenberger B.A., O’Laughlin M., Magrini V., Demeter R.T., Duncavage E.J. (2016). A common founding clone with TP53 and PTEN mutations gives rise to a concurrent germ cell tumor and acute megakaryoblastic leukemia. Mol. Case Stud..

[118] Chu J., Shi Z., Jiao Y., Han Z., Dou Q., Ye J., Cui X. (2018). B-cell lymphoma 2 ovarian killer suppresses testicular cancer cell malignant behavior, but plays a role in platinum resistance. Anticancer Drugs.

[119] Sano M., Nakanishi Y., Yagasaki H., Honma T., Oinuma T., Obana Y., Suzuki A., Nemoto N. (2005). Overexpression of anti-apoptotic Mcl-1 in testicular germ cell tumours. Histopathology.

[120] Chresta C.M., Masters J.R., Hickman J.A. (1996). Hypersensitivity of human testicular tumors to etoposide-induced apoptosis is associated with functional p53 and a high Bax:Bcl-2 ratio. Cancer Res..

[121] Burger H., Nooter K., Boersma A.W., Kortland C.J., Stoter G. (1998). Expression of p53, Bcl-2 and Bax in cisplatin-induced apoptosis in testicular germ cell tumour cell lines. Br. J. Cancer.

[122] Baltaci S., Orhan D., Turkolmez K., Yesilli C., Beduk Y., Tulunay O. (2001). P53, bcl-2 and bax immunoreactivity as predictors of response and outcome after chemotherapy for metastatic germ cell testicular tumours. BJU Int..

[123] Noel E.E., Yeste-Velasco M., Mao X., Perry J., Kudahetti S.C., Li N.F., Sharp S., Chaplin T., Xue L., McIntyre A. (2010). The association of CCND1 overexpression and cisplatin resistance in testicular germ cell tumors and other cancers. Am. J. Pathol..

[124] Costa A.L., Lobo J., Jeronimo C., Henrique R. (2017). The epigenetics of testicular germ cell tumors: Looking for novel disease biomarkers. Epigenomics.

[125] Koul S., McKiernan J.M., Narayan G., Houldsworth J., Bacik J., Dobrzynski D.L., Assaad A.M., Mansukhani M., Reuter V.E., Bosl G.J. (2004). Role of promoter hypermethylation in Cisplatin treatment response of male germ cell tumors. Mol. Cancer.

[126] Markulin D., Vojta A., Samarzija I., Gamulin M., Beceheli I., Jukic I., Maglov C., Zoldos V., Fucic A. (2017). Association between RASSF1A Promoter Methylation and Testicular Germ Cell Tumor: A Meta-analysis and a Cohort Study. Cancer Genom. Proteom..

[127] Ellinger J., Albers P., Perabo F.G., Muller S.C., von Ruecker A., Bastian P.J. (2009). CpG island hypermethylation of cell-free circulating serum DNA in patients with testicular cancer. J. Urol..

[128] Martinelli C., Lengert A.V.H., Carcano F.M., Silva E.C.A., Brait M., Lopes L.F., Vidal D.O. (2017). MGMT and CALCA promoter methylation are associated with poor prognosis in testicular germ cell tumor patients. Oncotarget.

[129] Beyrouthy M.J., Garner K.M., Hever M.P., Freemantle S.J., Eastman A., Dmitrovsky E., Spinella M.J. (2009). High DNA methyltransferase 3B expression mediates 5-aza-deoxycytidine hypersensitivity in testicular germ cell tumors. Cancer Res..

[130] Wongtrakoongate P., Li J., Andrews P.W. (2014). Aza-deoxycytidine induces apoptosis or differentiation via DNMT3B and targets embryonal carcinoma cells but not their differentiated derivatives. Br. J. Cancer.

[131] Biswal B.K., Beyrouthy M.J., Hever-Jardine M.P., Armstrong D., Tomlinson C.R., Christensen B.C., Marsit C.J., Spinella M.J. (2012). Acute hypersensitivity of pluripotent testicular cancer-derived embryonal carcinoma to low-dose 5-aza deoxycytidine is associated with global DNA Damage-associated p53 activation, anti-pluripotency and DNA demethylation. PLoS ONE.

[132] Wermann H., Stoop H., Gillis A.J., Honecker F., van Gurp R.J., Ammerpohl O., Richter J., Oosterhuis J.W., Bokemeyer C., Looijenga L.H. (2010). Global DNA methylation in fetal human germ cells and germ cell tumours: Association with differentiation and cisplatin resistance. J. Pathol..

[133] Singh R., Fazal Z., Corbet A.K., Bikorimana E., Rodriguez J.C., Khan E.M., Shahid K., Freemantle S.J., Spinella M.J. (2019). Epigenetic Remodeling through Downregulation of Polycomb Repressive Complex 2 Mediates Chemotherapy Resistance in Testicular Germ Cell Tumors. Cancers (Basel).

[134] Hsieh T.H., Liu Y.R., Chang T.Y., Liang M.L., Chen H.H., Wang H.W., Yen Y., Wong T.T. (2018). Global DNA methylation analysis reveals miR-214-3p contributes to cisplatin resistance in pediatric intracranial nongerminomatous malignant germ cell tumors. Neuro-Oncology.

[135] Liu L., Lian J., Zhang H., Tian H., Liang M., Yin M., Sun F. (2013). MicroRNA-302a sensitizes testicular embryonal carcinoma cells to cisplatin-induced cell death. J. Cell Physiol..

[136] Kondrashova O., Topp M., Nesic K., Lieschke E., Ho G.Y., Harrell M.I., Zapparoli G.V., Hadley A., Holian R., Boehm E. (2018). Methylation of all BRCA1 copies predicts response to the PARP inhibitor rucaparib in ovarian carcinoma. Nat. Commun..

[137] Levine A.J., Berger S.L. (2017). The interplay between epigenetic changes and the p53 protein in stem cells. Genes Dev..

[138] Huang J., Perez-Burgos L., Placek B.J., Sengupta R., Richter M., Dorsey J.A., Kubicek S., Opravil S., Jenuwein T., Berger S.L. (2006). Repression of p53 activity by Smyd2-mediated methylation. Nature.

[139] Shi X., Kachirskaia I., Yamaguchi H., West L.E., Wen H., Wang E.W., Dutta S., Appella E., Gozani O. (2007). Modulation of p53 function by SET8-mediated methylation at lysine 382. Mol. Cell.

[140] Zhu J., Dou Z., Sammons M.A., Levine A.J., Berger S.L. (2016). Lysine methylation represses p53 activity in teratocarcinoma cancer cells. Proc. Natl. Acad. Sci. USA.

[141] Dorssers L.C.J., Gillis A.J.M., Stoop H., van Marion R., Nieboer M.M., van Riet J., van de Werken H.J.G., Oosterhuis J.W., de Ridder J., Looijenga L.H.J. (2019). Molecular heterogeneity and early metastatic clone selection in testicular germ cell cancer development. Br. J. Cancer.

[142] Barrett M.T., Lenkiewicz E., Malasi S., Stanton M., Slack J., Andrews P., Pagliaro L., Bryce A.H. (2019). Clonal analyses of refractory testicular germ cell tumors. PLoS ONE.

[143] Necchi A., Bratslavsky G., Corona R.J., Chung J.H., Millis S.Z., Elvin J.A., Vergilio J.A., Suh J., Ramkissoon S., Severson E. (2020). Genomic Characterization of Testicular Germ Cell Tumors Relapsing After Chemotherapy. Eur. Urol. Focus.

[144] Peterson C.M., Buckley C., Holley S., Menias C.O. (2012). Teratomas: A multimodality review. Curr. Probl. Diagn. Radiol..

[145] David S., Andras F., Endre K., Balint K., Arpad K., Csaba P., Karoly S., Tamas T. (2017). More Cases of Benign Testicular Teratomas are Detected in Adults than in Children. A Clinicopathological Study of 543 Testicular Germ Cell Tumor Cases. Pathol. Oncol. Res..

[146] Anheuser P., Kranz J., Stolle E., Hoflmayer D., Buscheck F., Muhlstadt S., Lock G., Dieckmann K.P. (2019). Testicular epidermoid cysts: A reevaluation. BMC Urol..

[147] Moch H., Cubilla A.L., Humphrey P.A., Reuter V.E., Ulbright T.M. (2016). The 2016 WHO Classification of Tumours of the Urinary System and Male Genital Organs-Part A: Renal, Penile, and Testicular Tumours. Eur. Urol..

[148] Korkola J.E., Houldsworth J., Bosl G.J., Chaganti R.S. (2009). Molecular events in germ cell tumours: Linking chromosome-12 gain, acquisition of pluripotency and response to cisplatin. BJU Int..

[149] Van Echten J., Sleijfer D.T., Wiersema J., Schraffordt Koops H., Oosterhuis J.W., de Jong B. (1997). Cytogenetics of primary testicular nonseminoma, residual mature teratoma, and growing teratoma lesion in individual patients. Cancer Genet. Cytogenet..

[150] Einhorn L.H., Foster R.S. (2014). What are the indications for postchemotherapy retroperitoneal lymph node dissection?. Ann. Oncol..

[151] Donohue J.P., Fox E.P., Williams S.D., Loehrer P.J., Ulbright T.M., Einhorn L.H., Weathers T.D. (1994). Persistent cancer in postchemotherapy retroperitoneal lymph-node dissection: Outcome analysis. World J. Urol..

[152] Almstrup K., Lobo J., Morup N., Belge G., Rajpert-De Meyts E., Looijenga L.H.J., Dieckmann K.P. (2020). Application of miRNAs in the diagnosis and monitoring of testicular germ cell tumours. Nat. Rev. Urol..

[153] Nettersheim D., Arndt I., Sharma R., Riesenberg S., Jostes S., Schneider S., Holzel M., Kristiansen G., Schorle H. (2016). The cancer/testis-antigen PRAME supports the pluripotency network and represses somatic and germ cell differentiation programs in seminomas. Br. J. Cancer.

[154] Nettersheim D., Gillis A., Biermann K., Looijenga L.H., Schorle H. (2011). The seminoma cell line TCam-2 is sensitive to HDAC inhibitor depsipeptide but tolerates various other chemotherapeutic drugs and loss of NANOG expression. Genes Chromosomes Cancer.

[155] Skotheim R.I., Lind G.E., Monni O., Nesland J.M., Abeler V.M., Fossa S.D., Duale N., Brunborg G., Kallioniemi O., Andrews P.W. (2005). Differentiation of human embryonal carcinomas in vitro and in vivo reveals expression profiles relevant to normal development. Cancer Res..

[156] Honecker F., Rohlfing T., Harder S., Braig M., Gillis A.J., Glaesener S., Barett C., Bokemeyer C., Buck F., Brummendorf T.H. (2014). Proteome analysis of the effects of all-trans retinoic acid on human germ cell tumor cell lines. J. Proteom..

[157] Taylor-Weiner A., Zack T., O’Donnell E., Guerriero J.L., Bernard B., Reddy A., Han G.C., AlDubayan S., Amin-Mansour A., Schumacher S.E. (2016). Genomic evolution and chemoresistance in germ-cell tumours. Nature.

[158] Koster R., di Pietro A., Timmer-Bosscha H., Gibcus J.H., van den Berg A., Suurmeijer A.J., Bischoff R., Gietema J.A., de Jong S. (2010). Cytoplasmic p21 expression levels determine cisplatin resistance in human testicular cancer. J. Clin. Investig..

[159] Looijenga L.H., Stoop H., de Leeuw H.P., de Gouveia Brazao C.A., Gillis A.J., van Roozendaal K.E., van Zoelen E.J., Weber R.F., Wolffenbuttel K.P., van Dekken H. (2003). POU5F1 (OCT3/4) identifies cells with pluripotent potential in human germ cell tumors. Cancer Res..

[160] Moasser M.M., Motzer R.J., Khoo K.S., Lyn P., Murphy B.A., Bosl G.J., Dmitrovsky E. (1995). All-trans retinoic acid for treating germ cell tumors. In vitro activity and results of a phase II trial. Cancer.

[161] Pierpont T.M., Lyndaker A.M., Anderson C.M., Jin Q., Moore E.S., Roden J.L., Braxton A., Bagepalli L., Kataria N., Hu H.Z. (2017). Chemotherapy-Induced Depletion of OCT4-Positive Cancer Stem Cells in a Mouse Model of Malignant Testicular Cancer. Cell Rep..

[162] Paffenholz P., Pfister D., Matveev V., Heidenreich A. (2018). Diagnosis and management of the growing teratoma syndrome: A single-center experience and review of the literature. Urol. Oncol..

[163] Logothetis C.J., Samuels M.L., Trindade A., Johnson D.E. (1982). The growing teratoma syndrome. Cancer.

[164] Hiester A., Nettersheim D., Nini A., Lusch A., Albers P. (2019). Management, Treatment, and Molecular Background of the Growing Teratoma Syndrome. Urol. Clin. North Am..

[165] Michalski W., Jonska-Gmyrek J., Poniatowska G., Kucharz J., Stelmasiak P., Nietupski K., Sadowska M., Demkow T., Wiechno P. (2018). Testicular teratomas: A growing problem?. Med. Oncol..

[166] Scheckel C.J., Kosiorek H.E., Butterfield R., Ho T.H., Hilal T. (2019). Germ Cell Tumors with Malignant Somatic Transformation: A Mayo Clinic Experience. Oncol. Res. Treat..

[167] Necchi A., Colecchia M., Nicolai N., Piva L., Catanzaro M., Biasoni D., Torelli T., Stagni S., Paolini B., Milani A. (2011). Towards the definition of the best management and prognostic factors of teratoma with malignant transformation: A single-institution case series and new proposal. BJU Int..

[168] Giannatempo P., Pond G.R., Sonpavde G., Albany C., Loriot Y., Sweeney C.J., Salvioni R., Colecchia M., Nicolai N., Raggi D. (2016). Treatment and Clinical Outcomes of Patients with Teratoma with Somatic-Type Malignant Transformation: An International Collaboration. J. Urol..

[169] Colecchia M., Necchi A., Paolini B., Nicolai N., Salvioni R. (2011). Teratoma with somatic-type malignant components in germ cell tumors of the testis: A clinicopathologic analysis of 40 cases with outcome correlation. Int. J. Surg. Pathol..

[170] Dashti N.K., Jimenez R.E. (2017). Somatic-type malignancy in germ cell tumors. Pathology and Biology of Human Germ Cell Tumors.

[171] Zong X., Yang J.X., Zhang Y., Cao D.Y., Shen K., You Y., Guo L.N. (2019). Postchemotherapy sarcoma as a somatic-type malignancy derived from the gonadal yolk sac tumor in a patient with 46, XY pure gonadal dysgenesis. Onco Targets Ther..

[172] Singla N., Lafin J.T., Ghandour R.A., Kaffenberger S., Amatruda J.F., Bagrodia A. (2019). Genetics of testicular germ cell tumors. Curr. Opin. Urol..

[173] Chovanec M., De Giorgi U., Mego M. (2018). Immune-Related Concepts in Biology and Treatment of Germ-Cell Tumors. Adv. Urol..

[174] Chovanec M., Mardiak J., Mego M. (2019). Immune mechanisms and possible immune therapy in testicular germ cell tumours. Andrology.

[175] Fankhauser C.D., Curioni-Fontecedro A., Allmann V., Beyer J., Tischler V., Sulser T., Moch H., Bode P.K. (2015). Frequent PD-L1 expression in testicular germ cell tumors. Br. J. Cancer.

[176] Cierna Z., Mego M., Miskovska V., Machalekova K., Chovanec M., Svetlovska D., Hainova K., Rejlekova K., Macak D., Spanik S. (2016). Prognostic value of programmed-death-1 receptor (PD-1) and its ligand 1 (PD-L1) in testicular germ cell tumors. Ann. Oncol..

[177] Chovanec M., Cierna Z., Miskovska V., Machalekova K., Svetlovska D., Kalavska K., Rejlekova K., Spanik S., Kajo K., Babal P. (2017). Prognostic role of programmed-death ligand 1 (PD-L1) expressing tumor infiltrating lymphocytes in testicular germ cell tumors. Oncotarget.

[178] Adra N., Einhorn L.H., Althouse S.K., Ammakkanavar N.R., Musapatika D., Albany C., Vaughn D., Hanna N.H. (2018). Phase II trial of pembrolizumab in patients with platinum refractory germ-cell tumors: A Hoosier Cancer Research Network Study GU14-206. Ann. Oncol..

[179] Necchi A., Giannatempo P., Raggi D., Mariani L., Colecchia M., Fare E., Monopoli F., Calareso G., Ali S.M., Ross J.S. (2019). An Open-label Randomized Phase 2 study of Durvalumab Alone or in Combination with Tremelimumab in Patients with Advanced Germ Cell Tumors (APACHE): Results from the First Planned Interim Analysis. Eur. Urol..

[180] Mego M., Svetlovska D., Chovanec M., Reckova M., Rejlekova K., Obertova J., Palacka P., Sycova-Mila Z., De Giorgi U., Mardiak J. (2019). Phase II study of avelumab in multiple relapsed/refractory germ cell cancer. Investig. New Drugs.

[181] Oing C., Bokemeyer C. (2018). Biological basis and early clinical results of immunotherapy for cisplatin-resistant germ cell cancer. Curr. Opin. Urol..

[182] Zschabitz S., Lasitschka F., Jager D., Grullich C. (2016). Activity of immune checkpoint inhibition in platinum refractory germ-cell tumors. Ann. Oncol..

[183] Semaan A., Haddad F.G., Eid R., Kourie H.R., Nemr E. (2019). Immunotherapy: Last bullet in platinum refractory germ cell testicular cancer. Future Oncol..

[184] Oing C., Verem I., Mansour W.Y., Bokemeyer C., Dyshlovoy S., Honecker F. (2018). 5-Azacitidine Exerts Prolonged Pro-Apoptotic Effects and Overcomes Cisplatin-Resistance in Non-Seminomatous Germ Cell Tumor Cells. Int. J. Mol. Sci..

[185] Albany C., Hever-Jardine M.P., von Herrmann K.M., Yim C.Y., Tam J., Warzecha J.M., Shin L., Bock S.E., Curran B.S., Chaudhry A.S. (2017). Refractory testicular germ cell tumors are highly sensitive to the second generation DNA methylation inhibitor guadecitabine. Oncotarget.

[186] Steele N., Finn P., Brown R., Plumb J.A. (2009). Combined inhibition of DNA methylation and histone acetylation enhances gene re-expression and drug sensitivity in vivo. Br. J. Cancer.

[187] Roth B.J., Elson P., Sledge G.W., Einhorn L.H., Trump D.L. (1993). 5-Azacytidine (NSC 102816) in refractory germ cell tumors. Investig. New Drugs.

[188] Matei D., Ghamande S., Roman L., Alvarez Secord A., Nemunaitis J., Markham M.J., Nephew K.P., Jueliger S., Oganesian A., Naim S. (2018). A Phase I Clinical Trial of Guadecitabine and Carboplatin in Platinum-Resistant, Recurrent Ovarian Cancer: Clinical, Pharmacokinetic, and Pharmacodynamic Analyses. Clin. Cancer Res..

[189] Beyer U., Kronung S.K., Leha A., Walter L., Dobbelstein M. (2016). Comprehensive identification of genes driven by ERV9-LTRs reveals TNFRSF10B as a re-activatable mediator of testicular cancer cell death. Cell Death Differ..

[190] Nettersheim D., Jostes S., Fabry M., Honecker F., Schumacher V., Kirfel J., Kristiansen G., Schorle H. (2016). A signaling cascade including ARID1A, GADD45B and DUSP1 induces apoptosis and affects the cell cycle of germ cell cancers after romidepsin treatment. Oncotarget.

[191] Jostes S., Nettersheim D., Fellermeyer M., Schneider S., Hafezi F., Honecker F., Schumacher V., Geyer M., Kristiansen G., Schorle H. (2017). The bromodomain inhibitor JQ1 triggers growth arrest and apoptosis in testicular germ cell tumours in vitro and in vivo. J. Cell Mol. Med..

[192] Kurz L., Miklyaeva A., Skowron M.A., Overbeck N., Poschmann G., Becker T., Eul K., Kurz T., Schonberger S., Calaminus G. (2020). ARID1A Regulates Transcription and the Epigenetic Landscape via POLE and DMAP1 while ARID1A Deficiency or Pharmacological Inhibition Sensitizes Germ Cell Tumor Cells to ATR Inhibition. Cancers (Basel).

[193] Lobo J., Jeronimo C., Henrique R. (2020). Targeting the Immune system and Epigenetic Landscape of Urological Tumors. Int. J. Mol. Sci..

[194] Fenner M., Oing C., Dieing A., Gauler T., Oechsle K., Lorch A., Hentrich M., Kopp H.G., Bokemeyer C., Honecker F. (2019). Everolimus in patients with multiply relapsed or cisplatin refractory germ cell tumors: Results of a phase II, single-arm, open-label multicenter trial (RADIT) of the German Testicular Cancer Study Group. J. Cancer Res. Clin. Oncol..

[195] Oing C., Hentrich M., Lorch A., Glaser D., Rumpold H., Ochsenreither S., Richter S., Dieing A., Zschabitz S., Pereira R.R. (2020). Treatment of refractory germ-cell tumours with single-agent cabazitaxel: A German Testicular Cancer Study Group case series. J. Cancer Res. Clin. Oncol..

[196] Burger A.M., Double J.A., Newell D.R. (1997). Inhibition of telomerase activity by cisplatin in human testicular cancer cells. Eur. J. Cancer.

[197] Cressey T.R., Tilby M.J., Newell D.R. (2002). Decreased telomerase activity is not a reliable indicator of chemosensitivity in testicular cancer cell lines. Eur. J. Cancer.

[198] Skowron M.A., Vermeulen M., Winkelhausen A., Becker T.K., Bremmer F., Petzsch P., Schonberger S., Calaminus G., Kohrer K., Albers P. (2020). CDK4/6 inhibition presents as a therapeutic option for paediatric and adult germ cell tumours and induces cell cycle arrest and apoptosis via canonical and non-canonical mechanisms. Br. J. Cancer.

[199] Castillo-Avila W., Piulats J.M., Garcia Del Muro X., Vidal A., Condom E., Casanovas O., Mora J., Germa J.R., Capella G., Villanueva A. (2009). Sunitinib inhibits tumor growth and synergizes with cisplatin in orthotopic models of cisplatin-sensitive and cisplatin-resistant human testicular germ cell tumors. Clin. Cancer Res..

