# Supplementary material for: Cisplatin Resistance in Testicular Germ Cell Tumors: Current Challenges from Various Perspectives
Source: Cancers (Basel). 2020 Jun 17;12(6):1601. doi: 10.3390/cancers12061601 (PMC7352163; doi:10.3390/cancers12061601)
Supplement: Supplementary file 1 [file cancers-12-01601-s001.pdf]

cancers

Review

Cisplatin Resistance in Testicular Germ Cell Tumors: Current Challenges from Various Perspectives

João Lobo, Carmen Jerónimo and Rui Henrique

Table S1. Summary of some recent studies exploring cisplatin resistance in tumor models other than testicular germ cell tumors.

Tumor Model	Main Mechanism	Main Findings	Reference
Bladder Cancer	Uptake/Efflux/Detoxification, DNA repair, apoptosis, epigenetics, growth factors	Details the impact of extrinsic and intrinsic apoptosis pathways. Details epigenetic regulation of these pathways.	Broad review in [1]
Epithelial (i.e. non-GCT) Ovarian Cancer	DNA repair	Alterations of HR, NER, BER and MMR contribute to cisplatin sensitivity/resistance. Mutations in HR genes as biomarkers of response to PARPis	Broad review in [2]
	TLS	Overexpression of DNA polymerase eta contributes to cisplatin resistance	[3]
Lung cancer (SCLC)	Apoptosis	Sensitivity to cisplatin correlates with Bcl-2/Bim ratio. The BH3-mimetic ABT-263 sensitizes tumor cells to cisplatin and radiation	[4]
Lung cancer (NSCLC)	Epigenetics	Silencing the lncRNA UCA1 sensitizes tumor cells to cisplatin	[5]
	TLS	Overexpression of DNA polymerase eta predicts survival after treatment with cisplatin	[6]
Esophageal cancer	Epigenetics	miR-338-5p promoted cisplatin sensitivity in tumor cells, by targeting *FERMT2*	[7]
Head and neck cancer	Autophagy and microenvironment	CAFs contribute to cisplatin resistance by activating autophagy	[8]
Gastric cancer	EMT	The transcription factor eIF5A2 regulates cisplatin resistance by promoting EMT	[9]
Colorectal cancer	Apoptosis, inflammatory cytokines	The pro-inflammatory cytokine IL-17 promotes cisplatin resistance. Regulates apoptotic players Bax, Bcl-2	[10]
Breast cancer	p53 and inflammatory cytokines	The *TP63* isoform ΔNP63α (which counteracts p53 by promoting pro-survival signals) is induced by IL-1β and downregulates *ATM*, contributing to cisplatin resistance	[11]
Cervical cancer	Efflux, DNA repair, apoptosis, epigenetics, EMT, activation of stress response chaperones	Multifactorial perspective. Includes overview of several agents (including natural compounds) that antagonize cisplatin resistance	Broad review in [12]

Abbreviations: BER – base excision repair; CAF – cancer-associated fibroblast; EMT – epithelial-to-mesenchymal transition; HR – homologous recombination; lncRNA – long non-coding RNA; MMR –

mismatch repair; NER – nucleotide excision repair; NSCLC – non-small cell lung cancer; SCLC – small cell lung cancer; TLS – translesion synthesis.

Table S2. Review of the literature regarding growing teratoma syndrome with testicular origin.

Number of Patients	Age	Pre-CT Histology	Testicular Tumor laterality	Pre-CT Tumor size (cm)	Staging	Presentation	TM Before CT	Topography and Size of GTS Masses	Treatments Performed	Complications	Patient Outcome / Follow-up (months)	Relevant Details	Author (Ref.)
1	20y	Metastases: imTE	Left	Testis: 10x7x8; Metastases: N/A ("entire abdomen and pelvis + neck")	N/A; IGCCCG good risk	Testicular mass + abdominal distension + leg swelling (DVT)	Elevated	"Entire abdomen and pelvis" + neck (8cm)	3BEP→Orchiectomy along with radical Resection + LND	Seroma (resolved)	ANED (8)	Orchiectomy only after CT	Nassiri, 2019 [13]
1	19y	Testis: mixed	Left	N/A	III (lung metastases)	Testicular mass + constitutional symptoms	N/A	Lung + hilar, N/A	Orchiectomy→3BEP+3TIP →2 Resections	None	ANED (12)	-	Tanaka, 2018 [14]
22	Median 28y (IQR 22-34)	Testis: 16 Mixed, 2 EC, 2 YST, 2 TE	N/A	Testis: median 4.8 (IQR 1.9-9.4)	16 II, 5 III, 1 N/A	N/A	Elevated	Median 6 (IQR 4-12.2)	Orchiectomy→CT→RPLND	Adjunctive surgeries (n=4); rapid growth needing CT interruption (n=1); grade III/IV post-operative complications (n=4)	Disease relapse (n=2); DFD (n=1; 9 after RPLND); median 25 (IQR 8-47)	Only patients undergoing post-CT RPLND	Paffenholz and Pfister, 2018 [15]

1	36y	Testis: TE with ST cells	Left	Testis: 2; paratracheal nodes: 1; lombo-aortic nodes: 2	pT1	Testicular pain	Elevated β-HCG	Abdominal, 4.5; Mediastinal: 2; Cervical: 5	Orchiectomy→2BEP→RPLND→3 Resections	Horner syndrome	Disease relapse (GTS 3, 6, 8 after RPLND); ANED (48)	-	Priod, 2017 [16]
1	PT:47y; GTS: 51y	Testis: Mixed (YST, mTE, EC)	N/A	Testis: 10	I	Shortness of breath	Normal	Abdominal (19x18)+ Chest (14x12) + Thigh (16x10)	Orchiectomy→BEP+VIP→RPLND+2 Resections	None	Disease relapse (GTS 48 after orchiectomy); ANED (24)	-	Gange, 2016 [17]
2	PT: 23y; GTS: 24y	Testis: Mixed (TE, CH, EC)	Right	N/A	T2 (IIC); IGCCCG intermediate	N/A	N/A	Abdominal, 21	Orchiectomy→4BEP→Resection	None	Disease relapse (GTS 12 after orchiectomy); ANED (10)	-	Scavuzzo, 2014 [18]
	28y	Testis: Mixed (TE, EC)	Left		IIIA		Elevated AFP, LDH and β-HCG	Abdominal, 30	Orchiectomy→3BEP→Resection		Disease relapse (GTS 6 after orchiectomy); ANED (8)		
1	27y	N/A	Left	N/A	N/A	N/A	N/A	Abdominal, cardiac (right ventricle)	CT→Orchiectomy→ 2 Resections	Mild tricuspid regurgitation	ANED (24)	Orchiectomy only after CT; tricuspid valve	Roubelakis, 2014 [19]

												repair technique after tumor excision	
15	Median 23y (range: 16-39)	Testis: NS in 12, SE in 2, “hemorrhagic mass” in 1	N/A	N/A	8 II, 7 III	N/A	N/A	Retroperitoneal, median 7 (range 4-25); non-retroperitoneal in 4	Orchiectomy→CT→RPLND	2 major (Clavien ≥III); 5 additional procedures performed	ANED (8, range 1-64)	mTE in only 40% patients at orchiectomy	Lee, 2014 [20]

Abbreviations: AFP: alpha fetoprotein; ANED: alive with no evidence of disease; β-HCG: human chorionic gonadotropin subunit β; CH: choriocarcinoma; CT: chemotherapy; DFD: died from disease; DVT – deep venous thrombosis; EC: embryonal carcinoma; GTS – growing teratoma syndrome; IGCCCG: International Germ Cell Cancer Collaborative Group; imTE: immature teratoma; IQR: interquartile range; LDH: lactate dehydrogenase; LND: lymph-node dissection; mTE: mature teratoma; N/A – not available; NS – non-seminoma; PT: primary tumor; RPLND: retroperitoneal lymph-node dissection; SE – seminoma; TM: tumor markers; YST: yolk sac tumor

Table S3 – Review of the literature regarding growing teratoma syndrome with ovarian origin.

Patients (N)	Age	Histology (and Grade of NmTE)	Ovarian tumor laterality	Pre-CT Tumor Size (cm)	Staging	Presentation	TM Before CT	Topography and Size of GTS Mass (cm)	Treatments Performed	Complications	Patient Outcome / Follow-up After Last Treatment (months)	Relevant Details	Author (Ref.)
2	21y	Ovary: Mixed	Left	13x7	N/A	Abdominal distension	Elevated AFP	Adnexal, 35x20	3BEP→Resection	None	ANED (24)	-	Saba, 2018 [21]
	23y	Ovary: Mixed (imTE + YST)	Right	N/A		N/A	N/A	Adnexal, 7x4	3BEP→Resection	None	ANED (6)		
1	PT: 35y; GTS: 57y	Ovary: imTE	N/A	N/A	N/A	Asymptomatic	Normal	Pelvic + Hepatic (recurrence)	TH+BSO→5MTX/VP16→Resection	None	GTS 264 after 1st treatment; ANED (18)	Somatic-type malignancy in liver metastasis (follicular carcinoma) 22 years after primary tumor resection	Shannon, 2017 [22]
1	12y	Ovary: imTE, grade III	Right	Pelvic implants: 3.5x1.5; chest implants: 2x1; diaphragm implants: 2.5	FIGO IIIC	Abdominal distension with mass, weight loss	Elevated AFP	Pelvic, 9x8; peritoneal implant next to liver: 7x7	Right SO→4BEP→Resection→Experimental protocol (temozolomide + tretinoin + sorafenib)	None	"Without new tumor growths"	Rupture of primary tumor and seeding	Rentea, 2017 [23]
1	13y	Ovary: imTE (grade II);	Right	N/A	N/A	Abdominal distension	Elevated AFP	Abdominal (peri-	Right SO→Left SO +	None	Disease relapse (3 after right SO; 2	Patient was initially	Johnson,

		pelvic mass: imTE + mTE					and LDH	hepatic/splenic)	TH→4BEP+2EP →Resection		weeks after CT); ANED (30)	under-staged (IA)	2017 [24]
1	14y	Ovaries: mTE (left) + imTE grade III (right)	Bilateral	12-13	IA	Abdominal distension and pelvic pain	Elevated AFP	Liver, 5x4; left ovary: 6x4	Right SO + Left cystectomy→4EP→ 2 Resections	None	GTS 6 and 15 after SO; ANED (11)	-	Yakut, 2016 [25]
6	Median 24 (range 17-38)	Ovaries: only imTE patients included (grade III, n=5; grade I, n=1)	N/A	N/A	IA (n=1); IIIA (n=2); IIIC (n=1); IV (n=2, liver and small bowel)	Pelvic pain and bloating	N/A	Pelvic + Abdominal (all patients); mediastinal (n=1); 16-23	Surgery→4BEP/EP→1 to 4 additional resections (complete resections in 5/6 patients)	None	GTS (median 20 after surgery, range 8-42; median 12 after CT, range 2-48); recurrence of GTS (n=3, 12-132 after 1st GTS episode); ANED (n=5, median 90, range 24-166) and AWD (n=1)	Crude incidence of GTS = 40% (6/15 patients with imTE receiving CT); disease stage associated with GTS risk	Nguyen, 2016 [26]
1	PT: 16y; GTS: 25y	Ovary: imTE	Right	Ovary: 40x25	N/A	Abdominal distension and pain	N/A	Abdominal, 29x24	Right O→4BEP→ Resection	None	GTS 108 after O; ANED (14)	Successful pregnancy	Li S, 2016 [27]
1	PT: 14y; GTS: 24y	Ovary: imTE, grade II	Right	N/A	N/A	Abdominal pain	N/A	Abdominal (liver + spleen), 19x16	Right O→ 3BEP → Resection	None	GTS 120 after O; ANED (6)	-	Li X, 2016 [28]
1	10y	Ovaries/pelvic: imTE (biopsy)	N/A	N/A	N/A	Abdominal distension and pain	Elevated AFP	Abdominal, N/A	4BEP→Resection	None	ANED (6)	-	Kataria, 2016 [29]
1	37y	Ovary: imTE, grade III; pelvic recurrence: imTE + mTE	Left	N/A	N/A	N/A	Elevated AFP	Abdominal/pelvic + Liver, N/A	Left SO→ Resection →4BEP→ Resection	None	Disease relapse (4 after SO); GTS 9 after SO; ANED (10)	-	Soufi, 2015 [30]

1	PT: 20y; GTS: 21y	Ovary: imTE, grade III	Left	Ovary: 17; peritoneal implants: 0.5	FIGO IIIB	Abdominal distension	Elevated AFP	Abdominal, 5	Left SO→3BEP→ Resection (laparoscopic)	None	GTS 17 after SO; ANED (12)	Residual disease in SO; immature glial peritoneal implants	Shigeta, 2015 [31]
1	15y	Ovary: imTE, grade III	Left	Ovary: 25; implants: 8 and 5	N/A	Abdominal pain and asthenia	Elevated AFP	Retro-hepatic (12), cardiophrenic, pelvic (8)	Left SO→BEP+5TIP → Resections	None	ANED (N/A)	*Gliomatosis peritonei*	Portilla, 2015 [32]
1	15y	Ovary: imTE	Left	Ovarian/ Pelvic: 20x12; Peritoneal implants (7x6) and peri-hepatic (8x6)	FIGO III	Abdominal distension and pain	Elevated AFP	Peritoneal implants (7x3) and peri-hepatic (12x8)	Left SO→3BEP+5TIP → Resection	None	Disease progression (23 days after SO); ANED (5)	*Gliomatosis peritonei*	Ocamica, 2015 [33]
2	27y	Ovary: imTE, grade III	Left	Ovary: 19x15	IA	Abdominal and back pain	Normal	Pelvic, N/A	Left SO→3BEP+4EP → Resection → Surveillance	None	Disease relapse (8 after first resection); AWD (8)	*Gliomatosis peritonei;* viable pregnancy during treatment (after CT)	Merard, 2015 [34]
	22y	Ovary: imTE, grade II	Right	Ovary: 20x12	IIB	Neurologic / behavioral symptoms and abdominal distension	Elevated AFP	Liver (1st, 3.5); colon, pouch of Douglas and left ovary (2nd, 2)	Right SO→4BEP→ Resection→2BEP→ 2 Resections	Infertility	GTS 5 after SO; 2 after liver resection; "few months" after 2nd resection; ANED (108)	Paraneoplastic syndrome (anti-NMDA receptor encephaliti	

												s); rupture of primary tumor with seeding; residual disease in SO	
38	26y (range 8-41)	Ovaries: imTE + mTE (n=20); puré imTE (n=13); mixed (n=5); grade II (n=2), grade III (n=21)	N/A	N/A	I (n=10); II (n=2); III (n=13); N/A (n=13)	N/A	Normal (n=5); Elevated AFP (n=2); Elevated βHCG (n=2); N/A (n=16)	Peritoneum, wound, ovary	BEP (n=20), EP (n=2), BEP+EP (n=1), Other (n=7); fertility preserving surgery (n=20), 1-step surgery (n=25), 2-step surgery (n=13); complete resection in 25 patients	Clavien Dindo grade >2 complications (n=9)	Median follow-up of 73 (range 3-263); 10 patients (26%) with relapse (median time to relapse 45 months, range 2-132); 1 relapse (n=5), 2 relapses (n=2), 3 relapses (n=1); 1 DFD (60 after diagnosis), 1 DWD (heart disease)	Only patients with imTE included (19% with GTS); primary tumor rupture (n=4); 1 patient not fulfilling criteria (not treated with CT); 5 successful pregnancies; *gliomatosis peritonei* (n=10)	Bentivegna, 2015 [35]
1	21y	Ovary: imTE, grade III	Left	Ovary: 12x9.5	IC	Pelvic pain	Elevated AFP	Liver implant: 4x3; abdominal/ pelvic	Left SO→2EP → Resection→ EP→ Resection	None	Disease relapse (9 after 1st resection); ANED (36 after diagnosis)	Primary tumor rupture; progression during CT but increased	Pendlebury, 2014 [36]

												AFP – true case?	
1	PT: 29y; GTS: 30y	Ovary: “immature germ cell tumor”	Right	Ovary: 6x5.5	N/A	Abdominal pain and distension	Elevated AFP and βHCG	Liver and lung	Right SO→3BEP	None	GTS 12 after SO; ANED	-	Panda, 2014 [37]
1	PT: 12y; GTS: 30	Ovary: imTE	N/A	N/A	N+ (lymph nodes)	Abdominal mass, respiratory distress	N/A	Abdominal /pelvic, 40	TH+BSO→CT (incomplete)→Resection	Massive blood loss, respiratory complications	GTS 216 after surgery; ANED	-	Ohashi, 2014 [38]
5	PT: 20y (range 13-17)	Ovary: imTE (n=3) and YST (n=2)	N/A	N/A	III (n=2) and I (n=2)	N/A	Elevated AFP in all	Abdominal /pelvic (n=3), ovarian (n=2); 2.2-6.4, 3.3, 5.5	Surgery→BEP→ Surgery	None	GTS 29 after surgery (range 5-83); ANED or insufficient FUp	4/5 were asymptomatic at time of GTS diagnosis	Han, 2014 [39]
1	4y	Ovary: mixed (YST, TE, Dysg)	Left	17	N/A	Abdominal swelling, vomiting	Elevated AFP and βHCG	Retroperitoneal (multiple implants)	Resection→CT →Multiple resections	None	Disease relapse (4 after 1st resection, 7 after 2nd resection; 5 after 3rd resection); ANED	-	Daher, 2014 [40]

Abbreviations: AFP: alpha fetoprotein; ANED: alive with no evidence of disease; AWD: alive with disease; β-HCG: human chorionic gonadotropin subunit β; CT: chemotherapy; DFD: died from disease; DWD: died with disease; Dysg: dysgerminoma; FUp: follow-up; GTS – growing teratoma syndrome; imTE: immature teratoma; LDH: lactate dehydrogenase; mTE: mature teratoma; N/A – not available; O: oophorectomy; PT: primary tumor; SO: salpingo-oophorectomy; TH: total hysterectomy; TM: tumor markers; YST: yolk sac tumor

Table S4. – Review of the literature regarding growing teratoma syndrome with extragonadal origin.

Number of patients	Age	Primary Tumor Location	Primary Tumor Histology	Pre-CT Tumor Size (cm)	Staging	Presentation	TM Before CT	Topography and Size of GTS Masses	Treatments Performed	Complications	Patient Outcome / Follow-up after Last Treatment (months)	Relevant Details	Author (Ref.)
3 (♂)	24y	Med	imTE	13××13×16	N/A	Respiratory symptoms, chest pain	Elevated (S3)	Med, 12×15×19	3BEP→Resection	None	ANED (28)	-	Sachdeva et al, 2019 [41]
	21y		mTE	23×13×13			Elevated	Med, 25×18×15	3BEP+2EP→Resection	None	ANED (24)		
	22y		imTE	N/A				Med, 16×14×13	3BEP→2 resections	None	Disease relapse (7); ANED (16 after 2nd resection)		
1 (♂)	1m	Sacr	mTE	N/A	N/A	Detected in fetal US	N/A	Sacr, N/A	Resection→CT→Resection	Spinal cord compression	ANED (N/A)	-	Sertbas et al, 2019 [42]
1 (♂)	26y	Med	Mixed (SE, YST, TE)	N/A	N/A	Dyspnea, orthopnea and fever	Elevated	Med, 14×10	3BEP→2 resections	Massive bleeding (incomplete first resection)	Disease relapse (~12 after 1st resection); DFD (3 after 2nd resection)	Somatic-type malignancy in 2nd resection: angiosarcoma	Matsuoka et al, 2019 [43]
1 (♂)	PT: 23y;	Cranial (pineal)	N/A (biopsy non-	N/A	N/A	Headache, nausea,	Normal	Cranial, 2×1.5	RT→Resection	None	Disease relapse (108 after	No CT, but RT – true GTS?	Khoo B et al, 2017 [44]

	GTS: 32y		diagnostic)			poor balance					RT); ANED		
1 (♂)	PT: 51y; GTS: 69y	Med	mTE (first two resections)	5, 8 and 10×9	N/A	Mass	Normal in first two resections; elevated AFP and βHCG (third resection)	Med, 15×13	Resections at 51, 59 and 69 years→3BEP→ Resection	Severe respiratory distress needing mechanical ventilation	Disease relapse (96 after 1st resection and 120 after 2nd resection); ANED (54)	-	Hakiri et al, 2017 [45]
1 (♂)	37y	RP	Mi×ed (imTE, YST)	16 + 8	cT× N1 M1b S3 (IIIC)	Abdomina l distension and pain, weight loss	Elevated AFP, βHCG and LDH (S3)	Abdominal/p elvic, 10×8	Resection→4BEP → Resection	Severe myelodyspl asia secondary to therapy	DFD (5)	Klinefelter syndrome patient	Konheim , 2017 [46]
1 (♂)	5y	Cranial (pineal)	mTE (biopsy)	N/A	N/A	Gaze paresis and headache	Elevated AFP	Cranial, N/A	"Cisplatin-based CT" → Resections (incomplete) → interferonα-2b → Palbociclib → Thermoablations	Neurologic symptoms, central venous sinus thrombosis, respiratory distress	AWD (30 after diagnosis)	E×perimental drug Palbociclib (CDK inhibitor)	Schultz, 2015 [47]
1 (♂)	13y	Cranial (pineal)	Germinom a (biopsy)	3.3	N/A	Headache, gaze palsy and vomiting	Normal	Cranial, N/A	Open biopsy→3EP→RT → Resection →RT	None	Disease progressi on under CT (at 2nd course); ANED (9)	Biopsy not representative (mi×ed tumor?); report of another case of enlarging mTE, but not treated with CT	Oya, 2014 [48]

												(e×panding definition?)	
2 (♂)	12y	Cranial (pineal)	N/A	N/A	N/A	Headache, double vision	Elevated AFP (serum and CSF)	Cranial	Carboplatin/etoposide and ifosfamide/etoposide→partial resection→RT→Multiple resections along with chemotherapy→Final total resection and submyeloablative CT	None	Disease progression under CT after partial resection; ANED (36 after final treatment)	Successive partial resections; rhabdomyosarcoma in one of resections (somatic malignancy)	Glass, 2014 [49]
	7y	Cranial (pineal)	N/A	N/A	N/A	Diplopia, Parinaud's syndrome	Elevated AFP and β-HCG	Cranial	CT→partial resection→RT→>Resection	Precocious puberty; hemorrhage	DFD (4.5 after surgery)	"somatic malignancy in the form of carcinoma" at the second resection	

Abbreviations: AFP: alpha fetoprotein; ANED: alive with no evidence of disease; AWD: alive with disease; β-HCG: human chorionic gonadotropin subunit β; CSF: cerebral spinal fluid; CT: chemotherapy; DFD: died from disease; GTS – growing teratoma syndrome; imTE: immature teratoma; LDH: lactate dehydrogenase; Med: mediastinal; mTE: mature teratoma; N/A – not available; PT: primary tumor; RT: radiotherapy; RP: retroperitoneal; Sacr: sacral; SE – seminoma; TM: tumor markers; US: ultrasound; YST: yolk sac tumor

References

1. Drayton, R.M.; Catto, J.W. Molecular mechanisms of cisplatin resistance in bladder cancer. *Expert Rev. Anticancer* **2012**, *12*, 271–281, doi:10.1586/era.11.201.
2. Damia, G.; Broggini, M. Platinum Resistance in Ovarian Cancer: Role of DNA Repair. *Cancers (Basel)* **2019**, *11*, doi:10.3390/cancers11010119.
3. Srivastava, A.K.; Han, C.; Zhao, R.; Cui, T.; Dai, Y.; Mao, C.; Zhao, W.; Zhang, X.; Yu, J.; Wang, Q.E. Enhanced expression of DNA polymerase eta contributes to cisplatin resistance of ovarian cancer stem cells. *Proc. Natl. Acad. Sci. USA* **2015**, *112*, 4411–4416, doi:10.1073/pnas.1421365112.
4. Li, H.; Wang, H.; Deng, K.; Han, W.; Hong, B.; Lin, W. The ratio of Bcl-2/Bim as a predictor of cisplatin response provides a rational combination of ABT-263 with cisplatin or radiation in small cell lung cancer. *Cancer Biomark.* **2019**, *24*, 51–59, doi:10.3233/CBM-181692.
5. Liu, X.; Huang, Z.; Qian, W.; Zhang, Q.; Sun, J. Silence of lncRNA UCA1 rescues drug resistance of cisplatin to non-small-cell lung cancer cells. *J. Cell Biochem.* **2019**, *120*, 9243–9249, doi:10.1002/jcb.28200.
6. Ceppi, P.; Novello, S.; Cambieri, A.; Longo, M.; Monica, V.; Lo Iacono, M.; Giaj-Levra, M.; Saviozzi, S.; Volante, M.; Papotti, M.; et al. Polymerase eta mRNA expression predicts survival of non-small cell lung cancer patients treated with platinum-based chemotherapy. *Clin. Cancer Res.* **2009**, *15*, 1039–1045, doi:10.1158/1078-0432.CCR-08-1227.
7. Lin, W.C.; Chen, L.H.; Hsieh, Y.C.; Yang, P.W.; Lai, L.C.; Chuang, E.Y.; Lee, J.M.; Tsai, M.H. miR-338-5p inhibits cell proliferation, colony formation, migration and cisplatin resistance in esophageal squamous cancer cells by targeting FERMT2. *Carcinogenesis* **2019**, *40*, 883–892, doi:10.1093/carcin/bgy189.
8. Liao, J.K.; Zhou, B.; Zhuang, X.M.; Zhuang, P.L.; Zhang, D.M.; Chen, W.L. Cancer-associated fi broblasts confer cisplatin resistance of tongue cancer via autophagy activation. *Biomed. Pharm.* **2018**, *97*, 1341–1348, doi:10.1016/j.biopha.2017.11.024.
9. Sun, J.; Xu, Z.; Lv, H.; Wang, Y.; Wang, L.; Ni, Y.; Wang, X.; Hu, C.; Chen, S.; Teng, F., et al. eIF5A2 regulates the resistance of gastric cancer cells to cisplatin via induction of EMT. *Am. J. Transl. Res.* **2018**, *10*, 4269–4279.
10. Sui, G.; Qiu, Y.; Yu, H.; Kong, Q.; Zhen, B. Interleukin-17 promotes the development of cisplatin resistance in colorectal cancer. *Oncol. Lett.* **2019**, *17*, 944–950, doi:10.3892/ol.2018.9645.
11. Mendoza-Rodriguez, M.G.; Ayala-Sumuano, J.T.; Garcia-Morales, L.; Zamudio-Meza, H.; Perez-Yepez, E.A.; Meza, I. IL-1beta Inflammatory Cytokine-Induced TP63 Isoform NP63alpha Signaling Cascade Contributes to Cisplatin Resistance in Human Breast Cancer Cells. *Int. J. Mol. Sci.* **2019**, 20, doi:10.3390/ijms20020270.
12. Zhu, H.; Luo, H.; Zhang, W.; Shen, Z.; Hu, X.; Zhu, X. Molecular mechanisms of cisplatin resistance in cervical cancer. *Drug Des. Devel.* **2016**, *10*, 1885–1895, doi:10.2147/DDDT.S106412.
13. Nassiri, N.; Shah, A.; Daneshmand, S. Growing Teratoma Syndrome of Testicular Origin. *Urology* **2019**, *123*, 20–23, doi:10.1016/j.urology.2018.05.015.
14. Tanaka, K.; Toyokawa, G.; Tagawa, T.; Ijichi, K.; Haratake, N.; Hirai, F.; Oda, Y.; Maehara, Y. Successful Treatment of Growing Teratoma Syndrome of the Lung by Surgical Resection: A Case Report and Literature Review. *Anticancer Res.* **2018**, *38*, 3115–3118, doi:10.21873/anticanres.12571.
15. Paffenholz, P.; Pfister, D.; Matveev, V.; Heidenreich, A. Diagnosis and management of the growing teratoma syndrome: A single-center experience and review of the literature. *Urol. Oncol.* **2018**, *36*, 529 e523–529, doi:10.1016/j.urolonc.2018.09.012.
16. Priod, F.; Lorge, F.; Di Gregorio, M.; Dupont, M.V.; Nollevaux, M.C.; Faugeras, L.; Lawson, G.; Eucher, P.; D'Hondt, L. Recurrent Masses after Testicular Cancer: Growing Teratoma Syndrome. A Case Report and Review of the Literature. *Case Rep. Oncol.* **2017**, *10*, 910–915, doi:10.1159/000481397.
17. Gange, W.S.; Blackwell, R.H.; Biemer, J.; Barkan, G.A.; Picken, M.M.; Quek, M.L. Oligometastatic Growing Teratoma Syndrome: A Case for an Aggressive Surgical Approach. *Curr. Urol.* **2016**, *9*, 163–165, doi:10.1159/000447134.
18. Scavuzzo, A.; Santana Rios, Z.A.; Noveron, N.R.; Jimenez Rios, M.A. Growing teratoma syndrome. *Case Rep. Urol.* **2014**, *2014*, 139425, doi:10.1155/2014/139425.
19. Roubelakis, A.; Modi, A.; Ohri, S.K. Isolated tricuspid valve repair after metastatic tumor resection. *Ann. Thorac. Surg.* **2014**, *98*, 1447–1449, doi:10.1016/j.athoracsur.2013.12.057.

20. Lee, D.J.; Djaladat, H.; Tadros, N.N.; Movassaghi, M.; Tejura, T.; Duddalwar, V.; Daneshmand, S. Growing teratoma syndrome: clinical and radiographic characteristics. *Int. J. Urol.* **2014**, *21*, 905–908, doi:10.1111/iju.12466.
21. Saba, A.; Khan, R.S.; Ismail, H. Growing teratoma syndrome in ovarian germ cell tumours - a diagnostic challenge, two case reports. *J. Pak. Med. Assoc.* **2018**, *68*, 945–946.
22. Shannon, N.B.; Chan, N.H.L.; Teo, M.C.C. Recurrence of immature ovarian teratoma as malignant follicular carcinoma with liver and peritoneal metastasis 22 years after completion of initial treatment. *BMJ Case Rep.* **2017**, *2017*, doi:10.1136/bcr-2017-219665.
23. Rentea, R.M.; Varghese, A.; Ahmed, A.; Kats, A.; Manalang, M.; Dowlut-McElroy, T.; Hendrickson, R.J. Pediatric Ovarian Growing Teratoma Syndrome. *Case Rep. Surg.* **2017**, *2017*, 3074240, doi:10.1155/2017/3074240.
24. Johnson, L.R.; Sambasivan, S.; Nair, R.P.; Mony, R.P.; Sebastian, J.E.; Ahamed, I.M. Growing Teratoma Syndrome Following Treatment for Immature Teratoma of Ovary-A Case Report and Review of Literature. *J. Obs. Gynaecol. India.* **2017**, *67*, 295–298, doi:10.1007/s13224-016-0948-1.
25. Yakut, H.I.; Korkmaz, E.; Cetinkaya, N.; Baser, E.; Irkkan, C.; Gungor, T. Recurrent 'growing teratoma syndrome' in an adolescent girl with a history of ovarian immature teratoma: A case report. *J. Obs. Gynaecol.* **2016**, *36*, 845–847, doi:10.3109/01443615.2016.1168371.
26. Van Nguyen, J.M.; Bouchard-Fortier, G.; Ferguson, S.E.; Covens, A. How Common Is the Growing Teratoma Syndrome in Patients With Ovarian Immature Teratoma? *Int. J. Gynecol. Cancer* **2016**, *26*, 1201–1206, doi:10.1097/IGC.0000000000000751.
27. Li, S.; Liu, Z.; Dong, C.; Long, F.; Liu, Q.; Sun, D.; Gao, Z.; Wang, L. Growing Teratoma Syndrome Secondary to Ovarian Giant Immature Teratoma in an Adolescent Girl: A Case Report and Literature Review. *Med. (Baltim.)* **2016**, *95*, e2647, doi:10.1097/MD.0000000000002647.
28. Li, X.; Zhu, D.; Lv, L.I.; Yu, J. An uncommon recurrence of an immature teratoma: A case report. *Oncol. Lett.* **2016**, *11*, 2453–2456, doi:10.3892/ol.2016.4254.
29. Kataria, S.P.; Varshney, A.N.; Nagar, M.; Mandal, A.K.; Jha, V. Growing Teratoma Syndrome. *Indian J. Surg. Oncol.* **2017**, *8*, 46–50, doi:10.1007/s13193-016-0568-3.
30. 10Soufi, M.; Lupinacci, R.M.; Godiris-Petit, G.; Vignot, S.; Genestie, C.; Menegaux, F.; Lefranc, J.P. Growing teratoma syndrome of the ovary presenting with liver metastasis: report of a case. *Eur. J. Gynaecol. Oncol.* **2015**, *36*, 473–476.
31. Shigeta, N.; Kobayashi, E.; Sawada, K.; Ueda, Y.; Yoshino, K.; Hori, Y.; Kimura, T. Laparoscopic excisional surgery for growing teratoma syndrome of the ovary: case report and literature review. *J. Minim. Invasive Gynecol.* **2015**, *22*, 668–674, doi:10.1016/j.jmig.2015.01.011.
32. Gomez-Portilla, A.; Bustamante, R.; Lopez de Heredia, E.; Lete, L.I.; Cuadra, M.; Dominguez, S. Growing teratoma syndrome: an unusual evolution of treated ovarian immature teratoma - a case report. *Eur. J. Gynaecol. Oncol.* **2016**, *37*, 729–731.
33. Lapuente-Ocamica, O.; Ugarte, L.; Cuadra, M.; Lopez-Picado, A.; Maestro, L.; Lete, I. Growing teratoma syndrome after ovarian inmature teratoma: a case report and review of the literature. *Clin. Exp. Obs. Gynecol.* **2016**, *43*, 905–910.
34. Merard, R.; Ganesan, R.; Hirschowitz, L. Growing Teratoma Syndrome: A Report of 2 Cases and Review of the Literature. *Int. J. Gynecol. Pathol.* **2015**, *34*, 465–472, doi:10.1097/PGP.0000000000000180.
35. Bentivegna, E.; Azais, H.; Uzan, C.; Leary, A.; Pautier, P.; Gonthier, C.; Genestie, C.; Balleyguier, C.; Lhomme, C.; Duvillard, P.; et al. Surgical Outcomes After Debulking Surgery for Intraabdominal Ovarian Growing Teratoma Syndrome: Analysis of 38 Cases. *Ann. Surg. Oncol.* **2015**, *22* (3), S964–970, doi:10.1245/s10434-015-4608-y.
36. Pendlebury, A.; Rischin, D.; Ireland-Jenkin, K.; Toner, G.C.; Grant, P. Ovarian Growing Teratoma Syndrome With Spuriously Elevated alpha-Fetoprotein. *J. Clin. Oncol.* **2015**, 33, e99–100, doi:10.1200/JCO.2013.49.8998.
37. Panda, A.; Kandasamy, D.; Sh, C.; Jana, M. Growing teratoma syndrome of ovary: avoiding a misdiagnosis of tumour recurrence. *J. Clin. Diagn. Res.* **2014**, *8*, 197–198, doi:10.7860/JCDR/2014/7407.3837.
38. Ohashi, N.; Imai, H.; Tobita, T.; Ishii, H.; Baba, H. Anesthetic management in a patient with giant growing teratoma syndrome: a case report. *J. Med. Case Rep.* **2014**, *8*, 32, doi:10.1186/1752-1947-8-32.

39. Han, N.Y.; Sung, D.J.; Park, B.J.; Kim, M.J.; Cho, S.B.; Kim, K.A.; Song, J.Y. Imaging features of growing teratoma syndrome following a malignant ovarian germ cell tumor. *J. Comput Assist. Tomogr.* **2014**, *38*, 551–557, doi:10.1097/RCT.0000000000000073.
40. Daher, P.; Riachy, E.; Khoury, A.; Raffoul, L.; Ghorra, C.; Rehayem, C. Growing teratoma syndrome: first case report in a 4-year-old girl. *J. Pediatr. Adolesc. Gynecol.* **2015**, *28*, e5–e7, doi:10.1016/j.jpag.2014.03.003.
41. Sachdeva, A.K.; Penumadu, P.; Kohli, P.; Dubashi, B.; Munuswamy, H. Growing teratoma syndrome in primary mediastinal germ cell tumor: our experience. *Asian Cardiovasc. Thorac. Ann.* **2019**, *27*, 98–104, doi:10.1177/0218492318823345.
42. Sertbas, I.; Karatay, M. Enlarging teratoma syndrome. *Childs Nerv. Syst.* **2019**, *35*, 369–372, doi:10.1007/s00381-018-3956-x.
43. Matsuoka, S.; Koyama, T.; Takeda, T.; Yamada, K.; Hyogotani, A.; Hamanaka, K.; Sekiguchi, N.; Koizumi, T. Development of angiosarcoma in a mediastinal non-seminomatous germ cell tumor that exhibited growing teratoma syndrome during chemotherapy. *Thorac. Cancer* **2019**, *10*, 111–115, doi:10.1111/1759-7714.12901.
44. Khoo, B.; Ramakonar, H.H.; Robbins, P.; Honeybul, S. Intracranial monodermal teratoma presenting with growing teratoma syndrome. *J. Surg. Case Rep.* **2017**, 2017, rjx038, doi:10.1093/jscr/rjx038.
45. Hakiri, S.; Kawaguchi, K.; Fukui, T.; Fukumoto, K.; Nakamura, S.; Yokoi, K. Metachronous Germ Cell Tumors of the Mediastinum. *Ann. Thorac. Surg.* 2017, 103, e525–e527, doi:10.1016/j.athoracsur.**2016**.11.067.
46. Konheim, J.A.; Israel, J.A.; Delacroix, S.E. Klinefelter Syndrome with Poor Risk Extragonadal Germ Cell Tumor. *Urol. Case Rep.* **2017**, *10*, 1–3, doi:10.1016/j.eucr.2016.09.006.
47. Schultz, K.A.; Petronio, J.; Bendel, A.; Patterson, R.; Vaughn, D.J. PD0332991 (palbociclib) for treatment of pediatric intracranial growing teratoma syndrome. *Pediatr. Blood Cancer* **2015**, *62*, 1072–1074, doi:10.1002/pbc.25338.
48. Oya, S.; Saito, A.; Okano, A.; Arai, E.; Yanai, K.; Matsui, T. The pathogenesis of intracranial growing teratoma syndrome: proliferation of tumor cells or formation of multiple expanding cysts? Two case reports and review of the literature. *Childs Nerv. Syst.* **2014**, *30*, 1455–1461, doi:10.1007/s00381-014-2396-5.
49. Glass, T.; Cochrane, D.D.; Rassekh, S.R.; Goddard, K.; Hukin, J. Growing teratoma syndrome in intracranial non-germinomatous germ cell tumors (iNGGCTs): a risk for secondary malignant transformation-a report of two cases. *Childs Nerv. Syst.* **2014**, *30*, 953–957, doi:10.1007/s00381-013-2295-1.
